# Current State of Knowledge in Microbial Degradation of Polycyclic Aromatic Hydrocarbons (PAHs): A Review

**DOI:** 10.3389/fmicb.2016.01369

**Published:** 2016-08-31

**Authors:** Debajyoti Ghosal, Shreya Ghosh, Tapan K. Dutta, Youngho Ahn

**Affiliations:** ^1^Environmental Engineering Laboratory, Department of Civil Engineering, Yeungnam UniversityGyeongsan, South Korea; ^2^Disasters Prevention Research Institute, Yeungnam UniversityGyeongsan, South Korea; ^3^Department of Microbiology, Bose InstituteKolkata, India

**Keywords:** biodegradation, polycyclic aromatic hydrocarbons (PAHs), bacteria, fungi, algae

## Abstract

Polycyclic aromatic hydrocarbons (PAHs) include a group of organic priority pollutants of critical environmental and public health concern due to their toxic, genotoxic, mutagenic and/or carcinogenic properties and their ubiquitous occurrence as well as recalcitrance. The increased awareness of their various adverse effects on ecosystem and human health has led to a dramatic increase in research aimed toward removing PAHs from the environment. PAHs may undergo adsorption, volatilization, photolysis, and chemical oxidation, although transformation by microorganisms is the major neutralization process of PAH-contaminated sites in an ecologically accepted manner. Microbial degradation of PAHs depends on various environmental conditions, such as nutrients, number and kind of the microorganisms, nature as well as chemical property of the PAH being degraded. A wide variety of bacterial, fungal and algal species have the potential to degrade/transform PAHs, among which bacteria and fungi mediated degradation has been studied most extensively. In last few decades microbial community analysis, biochemical pathway for PAHs degradation, gene organization, enzyme system, genetic regulation for PAH degradation have been explored in great detail. Although, xenobiotic-degrading microorganisms have incredible potential to restore contaminated environments inexpensively yet effectively, but new advancements are required to make such microbes effective and more powerful in removing those compounds, which were once thought to be recalcitrant. Recent analytical chemistry and genetic engineering tools might help to improve the efficiency of degradation of PAHs by microorganisms, and minimize uncertainties of successful bioremediation. However, appropriate implementation of the potential of naturally occurring microorganisms for field bioremediation could be considerably enhanced by optimizing certain factors such as bioavailability, adsorption and mass transfer of PAHs. The main purpose of this review is to provide an overview of current knowledge of bacteria, halophilic archaea, fungi and algae mediated degradation/transformation of PAHs. In addition, factors affecting PAHs degradation in the environment, recent advancement in genetic, genomic, proteomic and metabolomic techniques are also highlighted with an aim to facilitate the development of a new insight into the bioremediation of PAH in the environment.

## Introduction

**FIGURE 1:**
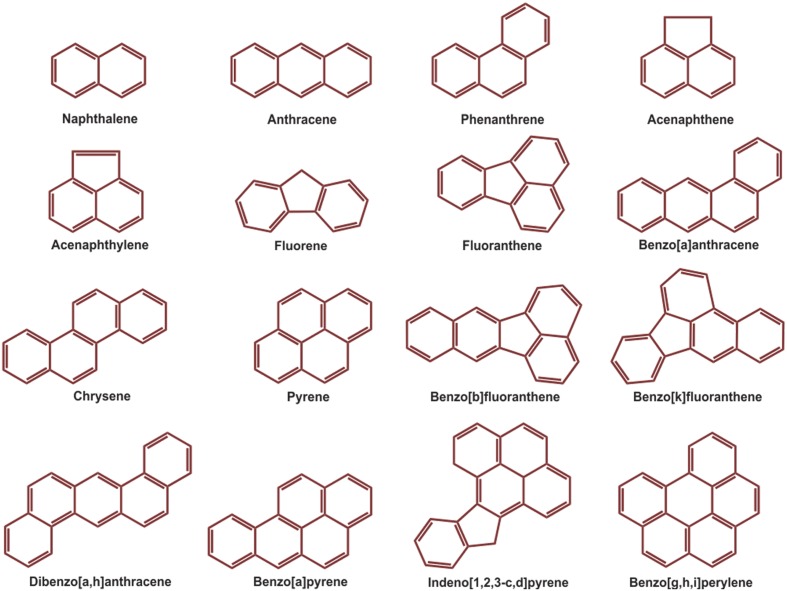
**Structure of the16 PAHs enlisted as priority pollutants by US EPA**.

Over the last few decades, with an increasing global awareness about the potential adverse effects of pollutants on public health and environment, remediation and renovation of environment contaminated with hazardous materials have received increasing attention. Among others, polycyclic aromatic hydrocarbons (PAHs) include a group of priority organic pollutants of significant concern due to their toxic, genotoxic, mutagenic and/or carcinogenic properties (WHO, 1983; Cerniglia, 1992; Mastrangelo et al., 1996; Schützendübel et al., 1999). PAHs are composed of fused aromatic rings in linear, angular, or cluster arrangements. Generally, the electrochemical stability, persistency, resistance toward biodegradation and carcinogenic index of PAHs increase with an increase in the number of aromatic rings, structural angularity, and hydrophobicity, while volatility tends to decrease with increasing molecular weight (Mackay and Callcott, 1998; Marston et al., 2001). The PAHs have a natural potential for bioaccumulation in various food chains, which make their presence in the environment quite alarming (Morehead et al., 1986; Xue and Warshawsky, 2005), and are therefore being considered as substances of potential human health hazards (Mastrangelo et al., 1996; Binkova et al., 2000; Marston et al., 2001; Xue and Warshawsky, 2005). On the basis of abundance and toxicity, 16 PAHs are already enlisted as priority environmental pollutants by the United States Environmental Protection Agency (US EPA), (Agency for Toxic Substances and Disease Registry [ATSDR], 1990; Liu et al., 2001) which are depicted in **Figure 1**. Various physical-chemical properties and some relevant information of 16 PAHs enlisted as priority pollutants by US EPA are depicted in **Table 1**.

**Table 1:** Physical-chemical properties and some relevant information of 16 PAHs enlisted as priority pollutants by US EPA.

Name	Molecular formula	Cas registry no.	Physical chemical properties				Toxicology			Biodegradation	
			B. Pt. (°C)	M.Pt (°C)	V.P. (mmHg at 25°C)	Solubility (mg/L)^a^	TEF^b^	IARC^c^	EPA^d^	Estimated half-lives (days)^e^	Measured half-lives (days)^f^
Naphthalene	C_10_H_8_	91-20-3	218	80.2	8.5 × 10^-2^	31	n.d.	2B	C	5.66	n.d.
Acenaphthene	C_12_H_10_	83-32-9	279	93.4	2.5 × 10^-3^	3.93	0.001	3	D	18.77	n.d.
Acenaphthylene	C_12_H_8_	208-96-8	280	91.8	6.68 × 10^-3^	1.93	0.001	n.c.	D	30.7	n.d.
Anthracene	C_14_H_10_	120-12-7	342	216.4	6.53 × 10^-6^	0.076	0.01	3	D	123	2.7
Phenanthrene	C_14_H_10_	85-01-8	340	100.5	1.2 × 10^-4^	1.20	0.001	3	D	14.97	5
Fluorene	C_13_H_10_	86-73-7	295	116-7	6.0 × 10^-4^	1.68–1.98	0.001	3	D	15.14	n.d.
Fluoranthene	C_16_H_10_	206-44-0	375	108.8	9.22 × 10^-6^	0.20–0.26	0.001	3	D	191.4	9.2
Benzo[*a*]anthracene	C_18_H_12_	56-55-3	438	158	4.11 × 10^-3^	0.010	0.1	2B	B2	343.8	> 182
Chrysene	C_18_H_12_	218-01-9	448	254	6.23 × 10^-9^	1.5 × 10^-3^	0.010	2B	B2	343.8	n.d.
Pyrene	C_16_H_10_	129-00-0	150.4	393	4.5 × 10^-6^	0.132	0.001	3	D	283.4	151
Benzo[*a*]pyrene	C_20_H_12_	50-32-8	495	179	5.49 × 10^-9^	3.8 × 10^-3^	1.0	1	B2	421.6	11
Benzo[*b*]fluoranthene	C_20_H_12_	205-99-2	481	168.3	5.0 × 10^-7^	0.0012	n.d.	2B	B2	284.7	n.d.
Benzo[*k*]fluoranthene	C_20_H_12_	207-08-9	480	215.7	9.7 × 10^-10^	7.6 × 10^-4^	0.1	2B	B2	284.7	n.d.
Dibenzo[a,h]anthracene	C_22_H_14_	53-70-3	524	262	9.55 × 10^-10^	5.0 × 10^-4^	n.d.	2A	B2	511.4	n.d.
Benzo[g,h,i]perylene	C_22_H_12_	191-24-2	500	277	1.0 × 10^-10^	2.6 × 10^-5^	n.d.	3	D	517.1	n.d.
Indenol[1,2,3-cd]pyrene	C_22_H_12_	193-39-5	536	161-3	1.25 × 10^-3^	0.062	n.d.	2B	B2	349.2	n.d.

*^a^(Mackay and Shiu, 1977). ^b^Toxic equivalent factor relatively to Benzo[a]pyrene (Chang et al., 2014). ^c^International Agency for Research on Cancer Classification Monographs Volume 1-111 updated 18 February 2015 (1, carcinogenic to humans; 2A, probably carcinogenic to humans; 2B, possibly carcinogenic to humans; 3, not classifiable as carcinogenic to humans; n.c., not classified). ^d^EPA carcinogenic classification: A, human carcinogenic; B1 and B2: probable human carcinogenic; C, possible human carcinogenic; D, not Classifiable as to human carcinogenicity; E, evidence of non-carcinogenicity for humans. ^e^Estimation using BioHCwin software v1.01 on EPI Suite software develop by (Howard et al., 2005). ^f^(Comber et al., 2012); n.d., not determined*.

In their pure chemical form, PAHs generally exist as colorless, white, or pale yellow-green solids having a faint, pleasant odor. They are basically non-polar organic compounds, characteristically composed of carbon and hydrogen atoms. Mostly incomplete combustion of organic materials like coal, tar, oil and gas, automobile exhaust, tobacco or smoked food, either during industrial and other human activities or during geothermal reactions associated with the production of fossil-fuels and minerals, result in PAH formation. In nature, they are formed during forest fires, volcanic eruptions, or by plant and bacterial reactions (Blumer, 1976; Wilson and Jones, 1993). Diverse types of combustion yield different distributions of PAHs in both relative amounts of individual PAHs as well as their isomers. In nature, they are formed during forest fires, volcanic eruptions, or by plant and bacterial reactions (Blumer, 1976; Wilson and Jones, 1993). Nevertheless, the anthropogenic input of PAHs to the environment far exceeds the natural sources (National Academy of Sceinces [NAS], 1971).

Polycyclic aromatic hydrocarbons are formed whenever organic substances are exposed to high temperatures (pyrolysis), and the composition of the products thus formed depends largely on the nature of the starting material as well as the transformation temperature (Blumer, 1976; Cerniglia, 1992). Fossil fuels also contain huge amounts of PAHs which are released into the environment during incomplete combustion or by accidental discharge during transport, use, or disposal of petroleum products or as a result of uncontrolled emissions (Cerniglia, 1992; Wilson and Jones, 1993; Johansson and van Bavel, 2003). PAHs are widely present as contaminants in air, soil, aquatic environments, sediments, surface water as well as in ground water (Huntley et al., 1993; Van Brummelen et al., 1996; Boxall and Maltby, 1997; Holman et al., 1999; Lim et al., 1999; Ohkouchi et al., 1999). Natural and anthropogenic sources of PAHs, in combination with global transport phenomena, result in their worldwide distribution and consequently, PAHs get dispersed from the atmosphere to vegetation, ultimately leading to bioaccumulation in various food chains (Edwards, 1983; Morehead et al., 1986; Wagrowski and Hites, 1997). Apart from biodegradation, the fate of PAH in nature varies depending on the environment, for example, in air, PAH can undergo photo-oxidation, whereas in the case of soil and water, they can undergo both photo-oxidation and chemical oxidation while some PAHs like naphthalene and alkyl naphthalene are partly lost by volatilization (Cerniglia, 1992).

The toxicity of PAH was first recognized in 1761 by John Hill, a physician who documented a high incidence of nasal cancer in tobacco snuff consumers (Cerniglia, 1984). The low-molecular-weight (LMW) PAHs (containing two or three aromatic rings) are acutely toxic while the high-molecular-weight (HMW) PAHs (containing four or more rings) are largely considered as genotoxic (Cerniglia, 1992; Mueller et al., 1996; Abdel-Shafy and Mansour, 2016). It is a known fact that PAH can covalently bind to DNA, RNA and proteins, but it is the amount of covalent interaction between PAHs and DNA that correlates best with carcinogenicity (Marston et al., 2001; Santarelli et al., 2008). In addition, the transformation products of some PAHs are more toxic than parent PAHs and can lead to critical cellular effects (Schnitz et al., 1993). In humans cytochrome P450 monooxygenase group of enzymes oxidize PAHs to epoxides, some of which are highly reactive (such as “bay-region” diol epoxides) and known as ultimate carcinogens. They can bind to DNA and have the ability to transform normal cells to malignant one (Milo and Casto, 1992; Schnitz et al., 1993; Marston et al., 2001). Studies have shown that processing food at high temperatures, like grilling or barbecuing result in high levels of PAHs in cooked meat and smoked fish (Morehead et al., 1986). The concern associated with PAHs further increased due to their ability to interfere with hormone metabolizing enzymes of the thyroid glands, and their adverse effects on reproductive as well as immune system (Veraldi et al., 2006; Oostingh et al., 2008).

Consequently, cleaning of such polluted places has been thought to be one of the most essential alternatives for restoring environmental damage. Several physical and chemical treatment methods including incineration, base-catalyzed de-chlorination, UV oxidation, fixation, solvent extraction etc. are already in practice (Norris et al., 1993; Gan et al., 2009), but have several drawbacks including cost, complexity, regulatory burden etc. Moreover, these conventional techniques, in many cases, do not destroy the contaminating compounds completely, but instead transfer them from one environment or form to another. In order to solve this burning problem, researchers have devised an efficient and eco-friendly clean-up technique known as bioremediation, which is being progressively refined to fight pollution. This technique utilizes and manipulates the detoxification abilities of living organisms to convert hazardous organic wastes including xenobiotics into harmless products, often carbon dioxide and water (Cerniglia and Heitkamp, 1989; Mueller et al., 1996; Bamforth and Singleton, 2005; Johnsen et al., 2005). Bioremediation addresses the limitations associated with most of the physicochemical processes by destroying many organic contaminants at reduced cost, under ambient conditions and thus, has now become a popular remedial alternative for pollutant removal including PAHs (Young and Cerniglia, 1996; Juhasz and Naidu, 2000; Kastner, 2000; Lovley, 2001; Andreoni and Gianfreda, 2007; Jorgensen, 2007; Megharaj et al., 2011; Abdel-Shafy and Mansour, 2016).

In addition, many natural habitats (e.g., aquifers, aquatic sediments) contaminated with a huge amount of aromatic pollutants are often anoxic. In these environments, the anaerobic degradation of aromatic compounds by microorganisms plays a major role in the removal of contaminants, recycling of carbon and sustainable development of the ecosystem. Reports on anaerobic biodegradation of PAHs are relatively recent, and only a limited number of preliminary studies have demonstrated the anaerobic degradation of PAHs including naphthalene, anthracene, phenanthrene, fluorene, acenaphthene and fluoranthene (Foght, 2008; Carmona et al., 2009; Mallick et al., 2011). However, detailed information on anaerobic degradation of PAHs under sulfate-reducing and nitrate-reducing conditions is scarce and very little is known about their degradation pathways, catabolic genes/enzymes and/or regulatory mechanisms, but this emerging field is ready to blossom in various aspects of biotechnological applications.

**FIGURE 2:**
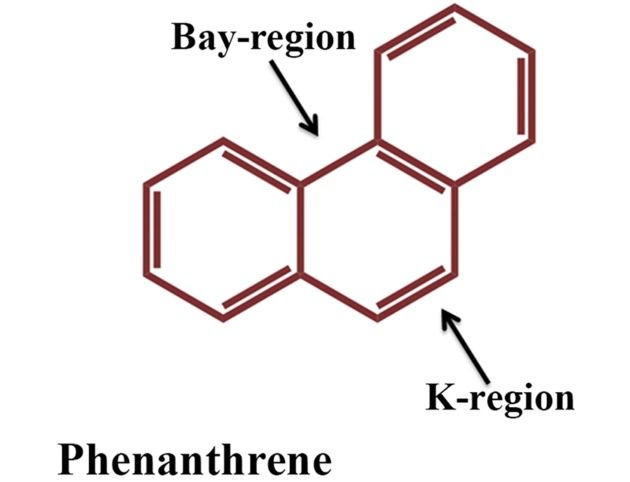
**Structure of phenanthrene, the simplest PAH containing bay and K region**.

Compared to HMW PAHs, LMW PAHs are reasonably more volatile and more soluble in water and consequently more susceptible to biodegradation (Pannu et al., 2003). LMW PAHs like naphthalene, anthracene and phenanthrene are widely present throughout the environment and designated as prototypic PAHs and serve as signature compounds to detect PAH contamination. Naphthalene represents the simplest PAH whereas the chemical structures of anthracene and phenanthrene are found in many carcinogenic PAHs (such as in benzo[a]pyrene, benz[a]anthracene etc.), and phenanthrene represents the smallest PAH to have both the bay and K regions (**Figure 2**). Thus, they are often used as a model substrate for studies on the metabolism of carcinogenic PAHs (Mallick et al., 2011) (and the references therein). In line with the advances in the knowledge of bacterial diversity within an ecosystem, many unique metabolic pathways on degradation of the PAHs are reported and scattered in literatures, which would collectively deepen the understanding on the range of catabolism as well as the biochemical and genetic diversities. Timely collective update of literature is most important to address current status of subjects related to our life and surrounding environment where the ongoing threat due to PAH-mediated pollution is no exception rather an issue that deserves top priority. The present review provides an overview of the current knowledge of microbial degradation/transformation of PAHs. Moreover, factors affecting biodegradation of PAHs, recent advancement in genetic, genomic, proteomic and metabolomic techniques and their application in bioremediation of PAHs have also been described.

## PAH Degradation by Bacteria and Halophilic Archaea

### Bacterial Catabolism of PAHs

Bacteria, which have evolved more than three billion years ago, have developed strategies for obtaining energy from virtually every compound and have been considered as nature’s ultimate scavengers. Because of their quick adaptability, bacteria have largely been used to degrade or remediate environmental hazards. Various bacteria have been found to degrade PAHs, in which degradation of naphthalene and phenanthrene has been most widely studied. Numerous unique metabolic pathways for the bacterial degradation of PAHs have been well documented in a number of excellent review articles (Cerniglia, 1992; Peng et al., 2008; Seo et al., 2009; Mallick et al., 2011). So, the biochemical pathways for bacterial degradation of PAHs will not be discussed in details in this communication. Nevertheless, there are two major strategies to degrade PAHs depending on the presence or absence of oxygen. In the aerobic catabolism of aromatics, the oxygen is not only the final electron acceptor but also a co-substrate for the hydroxylation and oxygenolytic ring cleavage of the aromatic ring. In contrast, the anaerobic catabolism of aromatic compounds uses a completely different strategy to attack the aromatic ring, primarily based on reductive reactions (Foght, 2008; Carmona et al., 2009). While the aerobic catabolism of aromatic compounds has been studied for several decades, the anaerobic degradation of aromatic compounds is a more recently discovered microbial capacity that still awaits a deeper understanding. However, anoxic conditions dominate in many natural habitats and contaminated sites (e.g., aquifers, aquatic sediments and submerged soils) where biodegradation is carried out by anaerobes using alternative final electron acceptors such as nitrate, sulfate or ferric ions (Foght, 2008; Carmona et al., 2009).

Principally, bacteria favor aerobic conditions for degradation of PAHs *via* oxygenase-mediated metabolism (involving either monooxygenase or dioxygenase enzymes). Usually, the first step in the aerobic bacterial degradation of PAHs is the hydroxylation of an aromatic ring *via* a dioxygenase, with the formation of a *cis*-dihydrodiol, which gets rearomatized to a diol intermediate by the action of a dehydrogenase. These diol intermediates may then be cleaved by intradiol or extradiol ring-cleaving dioxygenases through either an ortho-cleavage or meta-cleavage pathway, leading to intermediates such as catechols that are ultimately converted to TCA cycle intermediates (Evans et al., 1965; Cerniglia, 1992; Eaton and Chapman, 1992; Mueller et al., 1996; Mallick et al., 2011). Dioxygenase is a multicomponent enzyme generally consisting of reductase, ferredoxin, and terminal oxygenase subunits (Mallick et al., 2011). Bacteria can also degrade PAH *via* the cytochrome P450-mediated pathway, with the production of *trans*-dihydrodiols (Sutherland et al., 1990; Moody et al., 2004) or under anaerobic conditions, e.g., under nitrate-reducing conditions (Foght, 2008; Carmona et al., 2009).

Naphthalene degrading bacteria are ubiquitous in nature and there are enormous numbers of reports documenting the bacterial degradation of naphthalene including the elucidation of the biochemical pathways, enzymatic mechanisms and genetic regulations (Cerniglia, 1992; Peng et al., 2008; Seo et al., 2009; Lu et al., 2011; Mallick et al., 2011). The naphthalene catabolic genes present in the plasmid NAH7 in *Pseudomonas putida* G7 are well characterized (Simon et al., 1993). In the plasmid NAH7, the naphthalene catabolic genes (*nah*) are organized into two operons: the nal operon containing the genes for the upper pathway enzymes involved in conversion of naphthalene to salicylate, and the sal operon containing the genes for the lower pathway enzymes involved in the conversion of salicylate to pyruvate and acetaldehyde (Simon et al., 1993). The operons are positively regulated by a common regulator NahR, a LysR type of positive transcriptional regulator and are widely dispersed in bacteria. NahR is induced in presence of salicylate leading to high-level expression of the *nah* genes in bacteria (Yen and Gunsalus, 1985; Peng et al., 2008). There are several reports on the nucleotide sequences of genes encoding the upper pathway enzymes in different *Pseudomonas* strains, and the genes are more than 90% identical (Menn et al., 1993; Simon et al., 1993; Yang et al., 1994; Bosch et al., 2000; Peng et al., 2008). Moreover, in *Ralstonia* sp. U2, the naphthalene catabolic operon (nag) contains all of the upper pathway genes similar to that of the classical *nah* genes of *Pseudomonas* strains in the same order, with the exception of two extra genes named *nagG* and *nagH*, which are structural subunits of salicylate-5-hydroxylase enzyme required for the conversion of naphthalene to gentisate (Zhou et al., 2001). Additionally, in *Comamonas testeroni* strain GZ42, the naphthalene catabolic genes are similar to that found in *Ralstonia* sp. U2 (Goyal and Zylstra, 1997). The lower pathway genes for the naphthalene catabolism are also similar in different *Pseudomonas* strains like *P. putida* G7, NCIB9816-4, ND6, and *P. stutzeri* AN10 (Habe and Omori, 2003; Peng et al., 2008). In those organisms, lower pathway of naphthalene operon contains 11 genes present in the order *nahGTHINLOMKJY*, in which *nahY* represent a naphthalene chemotaxis gene. However, in AN10 and ND6 strains another salicylate hydroxylase gene (*nahW*) has been found to be present outside, but near to the sal operon.

Along with *Pseudomonas*, a high proportion of the PAH-degrading isolates belong to the sphingomonads, which comprise a physiologically versatile group within the Alphaproteobacteria: mainly *Sphingomonas, Sphingobium* and *Novosphingobium* that are frequently found as aromatic degraders. Species belonging to these genera show great catabolic versatility, capable of degrading a wide range of natural and xenobiotic compounds including HMW PAHs (Basta et al., 2005; Peng et al., 2008; Stolz, 2009; Vila et al., 2015). In a few reports, it has been shown that the catabolic versatility of sphingomonads relies on the large plasmids present in those organisms (Basta et al., 2005; Peng et al., 2008). However, the plasmid-encoded degradative genes have been found to be largely scattered, or they are not organized in coordinately regulated operons (Romine et al., 1999). It may be possible that this kind of ‘flexible’ gene organization i.e., different combinations of conserved gene clusters allows sphingomonads to adapt easily and proficiently to degrade various aromatic compounds including xenobiotics (Basta et al., 2005; Peng et al., 2008). Recently an illustrative report on the catabolic versatility of sphingomonads has shown that strain PNB, acting on diverse monoaromatic and polyaromatic compounds, contains seven sets of ring-hydroxylating oxygenases (RHO) with different substrate specificities (Khara et al., 2014). In general, it has been seen that mobile genetic elements (MGEs) like plasmids and transposons play a crucial role in the biodegradation of organic pollutants like PAHs. The presence of foreign compounds can often lead to the selection of mutant bacteria that are capable of metabolizing them. Apart from vertical gene transfer, aromatics catabolic genes are often harbored by MGEs that successfully disseminate the catabolic traits to phylogenetically diverse bacteria by horizontal gene transfer (Top and Springael, 2003; Nojiri et al., 2004).

**FIGURE 3:**
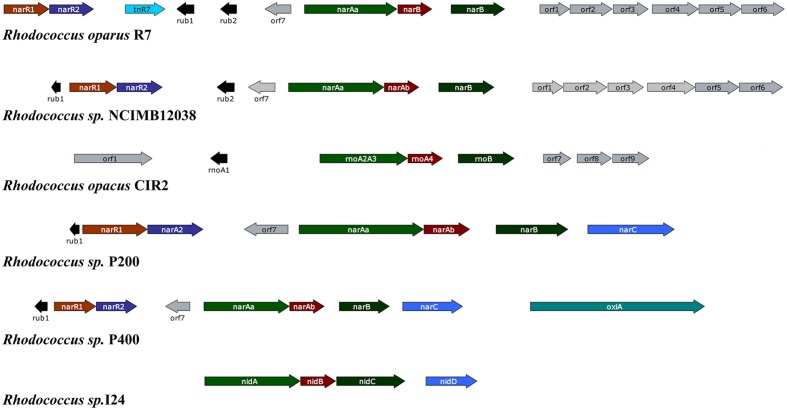
**Gene organization of the *nar* gene cluster of various naphthalene degrading *Rhodococcus* sp.** The respective *Rhodococcus* sp. along with the accession numbers of their corresponding naphthalene degrading gene cluster: *Rhodococcus opacus* R7 (accession no. DQ846881), *Rhodococcus* sp. NCIMB12038 (accession no. AF082663), *Rhodococcus opacus* CIR2 (accession no. AB024936), *Rhodococcus* sp. P200 (accession no. AY392424), *Rhodococcus* sp. P400 (accession no. AY392423), *Rhodococcus* sp. I24 (accession no. AF121905). The genes and their corresponding function of the gene products: *nar*R1- GntR-like regulator protein; *nar*R2- XylR-like regulator protein; *rub*1 and 2- Rubredoxin; *nar*Aa- Naphthalene dioxygenase large subunit; *nar*Ab- Naphthalene dioxygenase small subunit; *nar*B- Naphthalene dihydrodiol dehydrogenase (adapted from Di Gennaro et al., 2010).

Among PAHs degrading bacteria the genus *Rhodococcus* is very unique, having an enormous catabolic versatility. In contrast to *Pseudomonas* and other Gram-negative bacteria whose naphthalene catabolic genes are usually clustered, the Gram-positive *Rhodococcus* strains usually exhibit only three structural genes required for naphthalene degradation (*narAa*, *narAb* and *narB*) (Kulakov et al., 2005; Larkin et al., 2005). It has been seen that the nar region is not arranged into a single operon, and there are several homologous transcription units in different *Rhodococcus* strains separated by non-homologous sequences containing direct and inverted repeats (Larkin et al., 2005). The *narAa* and *narAb* genes codes for the α- and β- subunit of the naphthalene dioxygenase (NDO). Both subunits of NDO in *Rhodococcus* sp. strain NCIMB12038 showed only 30% amino acid identity to the corresponding *P. putida* NDO subunits. In addition, there is no gene in *Rhodococcu*s strains which is similar to the genes encoding the electron transport components reductase and ferredoxin of NDO in *Pseudomonas* strains (Kulakov et al., 2005; Larkin et al., 2005). Moreover, the *narA* and *narB* genes are transcribed as a single unit through different start sites, and their transcription is induced in the presence of naphthalene in contrast to salicylate-inducible naphthalene catabolic genes in *Pseudomonas* species. There are two putative regulatory genes (*narR1* and *narR2*, GntR-like transcriptional regulator) which are shown to be transcribed as a single mRNA in naphthalene-induced cells (Kulakov et al., 2005; Larkin et al., 2005). **Figure 3** represent the gene organization of the *nar* gene cluster of various naphthalene degrading *Rhodococcus* sp.

**FIGURE 4:**
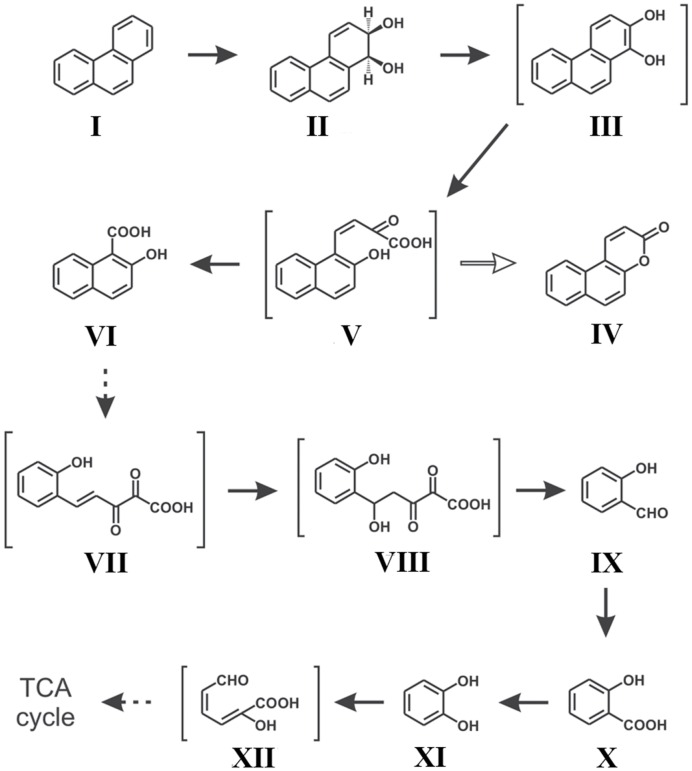
**Proposed pathway for the degradation of phenanthrene by *Ochrobactrum* sp. strain PWTJD.** The transient intermediates, which were not detected, are shown in parentheses. Filled arrows indicate mineralization; open arrows indicate a dead-end metabolite; and dotted arrows indicate multiple steps. Chemical designations: (I) phenanthrene; (II) *cis*-1,2-phenanthrenedihydrodiol; (III) 1,2-dihydroxyphenanthrene; (IV) 5,6-benzocoumarin; (V) *cis*-2-oxo-4-(20-hydroxynaphthyl)-but-3-enoic acid; (VI) 2-hydroxy-1-naphthoic acid; (VII) *trans*-2,3-dioxo-5-(20-hydroxyphenyl)- pent-4-enoic acid; (VIII) 2,3-dioxo-5-hydroxy-5-(20-hydroxyphenyl)-pentanoic acid; (XI) salicylaldehyde; (X) salicylic acid; (XI) catechol; (XII) 2-hydroxymuconaldehyde acid. Adapted from Ghosal et al. (2010).

Along with naphthalene, a number of reports on phenanthrene degradation by various Gram negative and Gram positive bacterial species have been reported (Peng et al., 2008; Seo et al., 2009; Mallick et al., 2011). In a study, Mallick et al. (2007) reported the degradation of phenanthrene by S*taphylococcus* sp. strain PN/Y, by initiating the dioxygenation specifically at the 1,2-position followed by *meta*-cleavage of phenanthrene-1,2-diol, leading to the formation of 2-hydroxy-1-naphthoic acid as the metabolic intermediate; while the *ortho*-cleavage could yield the naphthalene-1,2-dicarboxylic acid. Authors also reported that 2-hydroxy-1-naphthoic acid was metabolized by a *meta*-cleavage enzyme 2-hydroxy-1-naphthoate dioxygenase leading to the formation of *trans*-2,3-dioxo-5-(2′-hydroxyphenyl)-pent-4-enoic acid, a novel metabolite in the phenanthrene degradation pathway, and was subsequently degraded *via* salicylic acid and catechol (Mallick et al., 2007). Later on, Ghosal et al. (2010) reported the assimilation of phenanthrene by *Ochrobactrum* sp. strain PWTJD, isolated from municipal waste contaminated soil sample using phenanthrene as a sole source of carbon and energy. The strain PWTJD could also degrade phenanthrene *via* 2-hydroxy-1-naphthoic acid, salicylic acid and catechol. The strain PWTJD was found to utilize 2-hydroxy-1-naphthoic acid and salicylic acid as sole carbon sources, while the former was metabolized by a ferric-dependent meta-cleavage dioxygenase. In the lower pathway, salicylic acid was metabolized to catechol and was further degraded by catechol 2,3-dioxygenase to 2-hydroxymuconoaldehyde acid, ultimately leading to TCA cycle intermediates. The metabolic pathway involved in the degradation of phenanthrene by *Ochrobactrum* sp. strain PWTJD is shown in **Figure 4** (Ghosal et al., 2010). This was the first report of the complete degradation of a PAH molecule by Gram-negative *Ochrobactrum* sp. describing the involvement of the meta-cleavage pathway of 2-hydroxy-1-naphthoic acid in phenanthrene assimilation.

**FIGURE 5:**
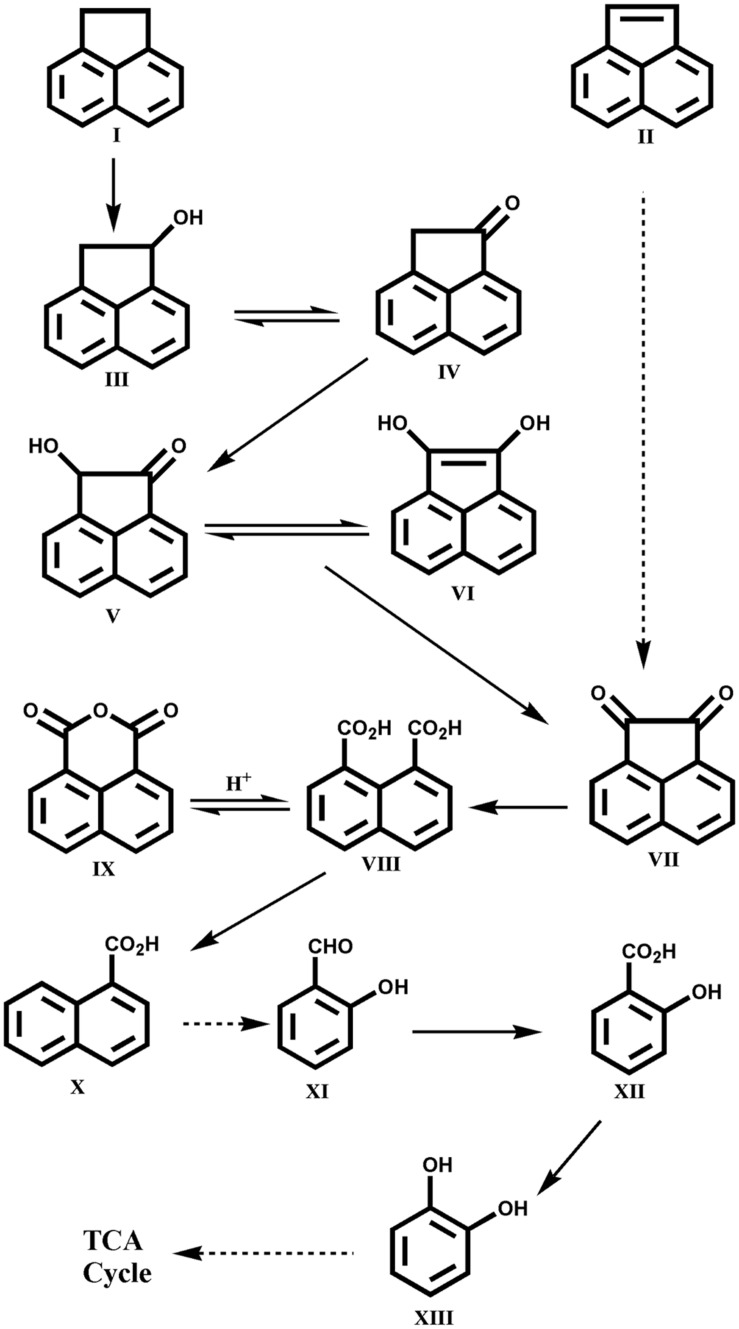
**Proposed pathway for the degradation of acenaphthene by *Acinetobacter* sp. strain AGAT-W.** Chemical designations: (I) acenaphthene; (II) acenaphthylene; (III) 1-acenaphthenol; (IV) 1-acenaphthenone; (V) 1-hydroxy-2-ketoacenaphthene; (VI) 1,2-dihydroxyacenaphthylene; (VII) acenaphthenequinone; (VIII) naphthalene-1,8-dicarboxylic acid; (IX) 1,8-naphthalic anhydride; (X) 1-naphthoic acid; (XI) salicylaldehyde; (XII) salicylic acid; (XIII) catechol. Adapted from Ghosal et al. (2013).

Other LMW PAHs like anthracene, fluorene, acenaphthene and acenaphthylene are also found in high quantities in PAH-polluted sites and various bacterial species have the capability to utilize these compounds individually as sole carbon and energy sources (Moody et al., 2001; Peng et al., 2008; Seo et al., 2009; Mallick et al., 2011). Lately, Ghosal et al. (2013) reported assimilation of acenaphthene and acenaphthylene by the *Acinetobacter* sp. strain AGAT-W, isolated from municipal waste contaminated soil sample using acenaphthene as a sole source of carbon and energy. The strain AGAT-W could degrade acenaphthene *via* 1-acenaphthenol, naphthalene-1,8-dicarboxylic acid, 1-naphthoic acid, salicylic acid and *cis*, *cis* muconic acid ultimately leading to TCA cycle intermediates. The metabolic pathways involved in the degradation of acenaphthene and acenaphthylene by *Acinetobacter* sp. strain AGAT-W is shown in **Figure 5** (Ghosal et al., 2013). This was the first report on the complete degradation of acenaphthene and acenaphthylene individually by strain AGAT-W belonging to the genus *Acinetobacter*.

Polycyclic aromatic hydrocarbons with more than three rings viz. pyrene, benzo[a] pyrene (Bap), are generally referred to as HMW PAHs (**Figure 1**), which are of significant environmental concern due to their long persistence and high toxicity as well as their mutagenic and/or carcinogenic properties (Cerniglia, 1992; Kanaly and Harayama, 2000; Peng et al., 2008; Seo et al., 2009). In the last few decades research on microbial degradation of HMW PAHs has advanced significantly and a number of HMW PAH-degrading isolates have been reported (Cerniglia, 1992; Kanaly and Harayama, 2000, 2010; Peng et al., 2008; Seo et al., 2009). Biodegradation of HMW PAHs by microorganisms is discussed adequately in many excellent reviews and the pathways for their degradation have also been depicted (Kanaly and Harayama, 2000; Peng et al., 2008; Seo et al., 2009). However, further investigations are prerequisite in several areas of HMW PAH biodegradation, namely, research related to the regulatory mechanisms of HMW PAH biodegradation, biodegradation of HMW PAH associated with other hydrocarbons in mixtures; and the interactions of complex microbial community during HMW PAH degradation can be exploited in more details. These will enrich our understanding on the microbial ecology of HMW PAH-degrading communities and the mechanisms by which HMW PAH biodegradation occur. In addition, the outcome will also help in predicting the environmental fate of these recalcitrant compounds and aid for the development of convenient as well as cost-effective bioremediation strategies in near future.

### Degradation of PAHs by Halophilic/Halotolerant Bacteria and Archaea

Environmental pollution due to anthropogenic activity has spread to all types of ecosystems and marine ecosystem is not excluded from this list. Hypersaline environments are regularly being polluted with organic pollutants including PAHs, through industrial and municipal eﬄuents. Industrial eﬄuents, specifically, petroleum industry where one of the main sources of contaminants in the waste waters are aromatic hydrocarbons including PAHs. Contamination and biodegradation in extreme environments like high salinity has been receiving increased attention in recent times (Oren et al., 1992; Margesin and Schinner, 2001a,b; Le Borgne et al., 2008; Bose et al., 2013; Cravo-Laureau and Duran, 2014; Elango et al., 2014; Fathepure, 2014; Genovese et al., 2014; Kappell et al., 2014; Kostka et al., 2014; Thomas et al., 2014; Torlapati and Boufadel, 2014). In addition, a survey of relevant literatures indicates that along with numerous biotechnological applications, halophilic microorganisms have more extended catabolic versatility than previously thought about (Margesin and Schinner, 2001b; Garcia et al., 2005; Smith et al., 2013; Kappell et al., 2014; Lamendella et al., 2014; Roling and Bodegom, 2014; Scott et al., 2014; Singh et al., 2014).

The biological treatment of industrial hypersaline waste waters and the bioremediation of polluted hypersaline environments are not possible with conventional microorganisms because they are incapable to function efficiently at salinities that of seawater or above. Thus, halophilic microorganisms are the best alternatives to overcome this problem (Oren et al., 1992; Garcia et al., 2005; Fathepure, 2014; Kostka et al., 2014). In the last decade there has been an increasing interest in the development and optimization of bioremediation processes *via* halophiles to deal with hypersaline environments that are contaminated with organic pollutants (Margesin and Schinner, 2001a; Mellado and Ventosa, 2003; Peyton et al., 2004; Garcia et al., 2005; Cui et al., 2008; Mnif et al., 2009; Zhao et al., 2009; Bonfa et al., 2011; Smith et al., 2013; Fathepure, 2014; Kappell et al., 2014; Lamendella et al., 2014; Singh et al., 2014; Thomas et al., 2014). Some halophiles that have shown PAHs degrading property are depicted in Supplementary Table S1. However, studies concerning the ability of this group of microbes to degrade PAHs are still in their infancy. Although in the last few years, a few studies on the degradation of PAHs by halophiles have been published. In this context, Gauthier et al. (1992) described the isolation of *Marinobacter hydrocarbonoclasticus* which can degrade various organic compounds including naphthalene (Gauthier et al., 1992). The presence of naphthalene dioxygenase, similar to those from *Pseudomonas* and *Burkholderia* spp. was reported in a naphthalene-degrading *Marinobacter* sp. strain NCE312 (Hedlund et al., 2001). Again, based on the analysis of the shoreline sand and rocks (Costa da Morte, northwestern Spain) affected by the Prestige oil spill, unveiled the high relative abundances of *Sphingomonadaceae* and *Mycobacterium* that could be associated with PAH degradation (Alonso-Gutierrez et al., 2009). In a similar study, Vila et al. (2010) reported the isolation of a marine microbial consortium from a beach polluted with the Prestige oil spill which is efficient in removing different hydrocarbon present in heavy fuel oil including three to five-ring PAHs (for example anthracene, fluoranthene, pyrene, benzo(a)anthracene, chrysene, and BaP) (Vila et al., 2010). In addition based on community dynamics analysis, it has been contemplated that Alphaproteobacteria (*Maricaulis* and *Roseovarius*), could be associated with the degradation of LMW PAHs, whereas Gammaproteobacteria (*Marinobacter* and *Methylophaga*), could be associated with the degradation of HMW PAHs. A similar bacterial community associated with the degradation of various organic compounds was found in a beach affected by the Deepwater Horizon oil spill (Kostka et al., 2011; Lamendella et al., 2014). Recently, Gallego et al. (2014) reported the isolation of a marine pyrene-degrading microbial consortium from a beach polluted by an oil spill and found that an uncultured *Gordonia* sp. is the key pyrene degrader in the consortium based on community structure and PAH ring-hydroxylating genes analyses (Gallego et al., 2014). Nevertheless, detailed information on the bioremediation of PAHs under high salinity by halophilic/halotolerant bacteria and archaea is still in its initial stage of exploration, but it is expected that this emerging field is ready to flourish in near future.

### Occurrence of PAH-Degrading Machinery in Diverse Bacterial/Halophilic Archaeal Genera

**FIGURE 6:**
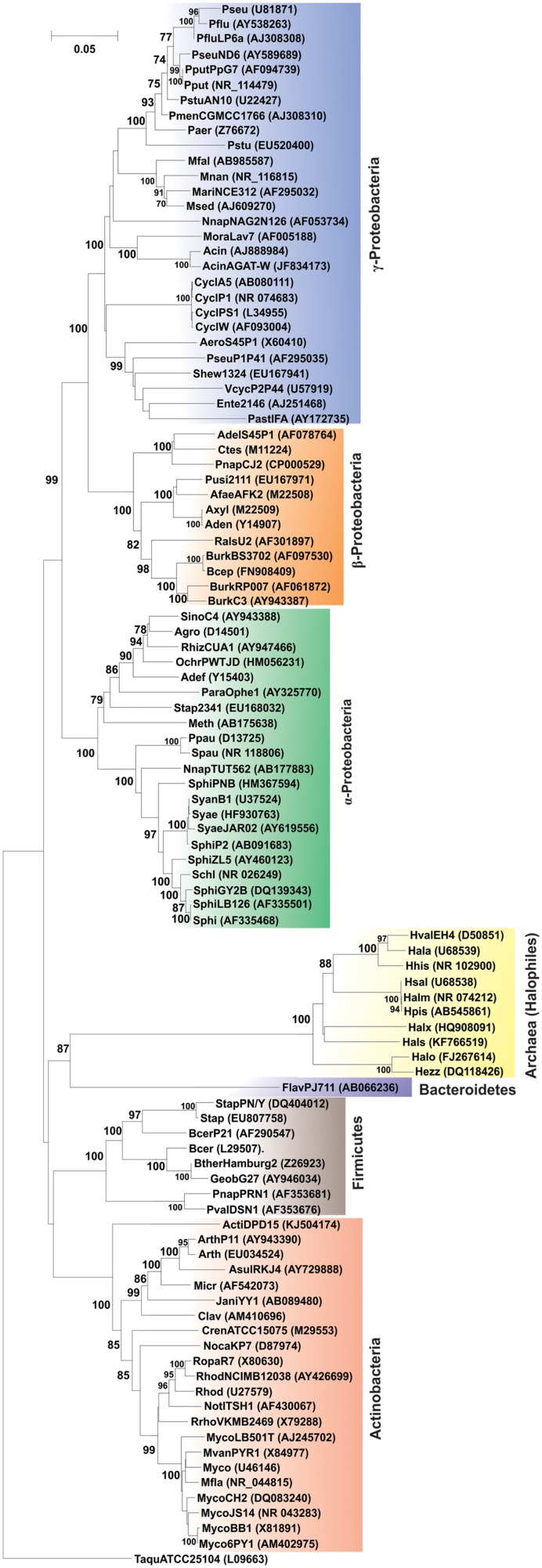
**Phylogenetic tree based on 16S rRNA gene sequence of organisms enlisted in Supplementary Table S1.** Organism designations are according to those given in Supplementary Table S1. However, for organisms devoid of 16S rRNA gene sequence information, sequence of representative type strain has been considered. Accession numbers are given within parentheses. 16S rRNA gene sequence of *Thermus aquaticus* strain ATCC 25104 was used as outgroup. Numbers at nodes indicate levels of bootstrap support based on neighbor-joining analysis of 100 resampled datasets. Bootstrap values below 70% are not shown. Bar represents 0.05 substitutions per nucleotide position.

The community analysis of indigenous microorganisms capable of degrading various aromatic compounds in diverse environments has been of great interest in the recent years (Watanabe et al., 2002; Brakstad and Lodeng, 2005; Gerdes et al., 2005; Yakimov et al., 2005; Coulon et al., 2007; Cui et al., 2008) and different strategies have been developed for the study of associated microbial communities (Widada et al., 2002). Previously it was thought that PAH degradation capabilities may be associated with certain genera or groups of bacteria independent of the origin of the source sample (Kastner et al., 1994; Mueller et al., 1997). But consequently, with time it has been reported that PAH-degrading bacteria are far more diverse (Widada et al., 2002; Andreoni et al., 2004; Cui et al., 2008; Hilyard et al., 2008). Currently, there are enormous reports of various bacterial as well as some archaeal genera capable of degrading PAHs. Supplementary Table S1 represents the bacterial/archaeal (halophilic) strains involved in the degradation of various PAHs. **Figure 6** illustrates the 16S rRNA gene-based phylogenetic analysis of all the bacterial/archaeal strains cited in Supplementary Table S1. It has been seen that bacterial/archaeal strains having PAHs catabolic property are distributed mainly in six major groups: Alphaproteobacteria, Betaproteobacteria, Gammaproteobacteria, Actinomycetes, Firmicutes and Archaea (Halophiles). It may be mentioned here that many strains listed in Supplementary Table S1 have not been cultured in laboratory. Some PAHs degrading isolates represent novel strains which are isolated from various geographically diverse habitats. Thus, all this information illustrate that PAH-degrading machinery is not confined to a few particular genus reported so far but is more likely to be distributed widely in the prokaryotic kingdom found in diverse geographical niches.

## Fungal Degradation of PAHs

The biodegradation of PAHs by fungi has been studied extensively in last several years and numerous fungal species have been reported to metabolize different PAHs (Cerniglia, 1992; Cerniglia and Sutherland, 2010). Most fungi cannot use PAHs as sole sources of carbon and energy; however, they may co-metabolize PAHs to a wide variety of oxidized products and sometimes to CO_2_. Bacterial PAHs degradation mainly involves dioxygenase enzymes and partially monooxygenase mediated reactions and the same is valid for algae. For example, the extent of dioxygenase vis-à-vis monooxygenase catalyzed transformation of naphthalene by *Mycobacterium* sp. was found to be in the ratio of around 25:1 (Kelley et al., 1990). On the other hand, fungal PAHs degradation mainly involves monooxygenase enzymes (Cerniglia and Sutherland, 2010) (and the references therein). However, the transformation of PAHs by fungi involves several enzymatic pathways and depends on the particular species and growth conditions. The fungi involved in PAHs biodegradation are mainly of two types- ligninolytic fungi or white-rot fungi (they have the ability to produce enzymes including lignin peroxidase (LiP), manganese peroxidase (MnP) and laccases to degrade the lignin in wood) and non-ligninolytic fungi (those who do not produce peroxidases or laccases but instead produce cytochrome P450 monooxygenase like enzymes) (Hofrichter, 2002; Tortella et al., 2005; Cerniglia and Sutherland, 2010; Li et al., 2010). Although, various ligninolytic fungi such as *Phanerochaete chrysosporium* and *Pleurotus ostreatus* can secrete both ligninolytic and non-ligninolytic type of enzymes, but it is uncertain to what extent each enzyme participates in the degradation of the PAHs (Bezalel et al., 1997). On the other hand, another type of ligninolytic fungi, known as brown-rot fungi, mainly produces hydrogen peroxide for degrading hemicelluloses and cellulose. Although, only limited data is available about PAH metabolism by brown rot fungi, some brown-rot fungi such as *Laetiporus sulphureus* and *Flammulina velutipes* have been shown to metabolize PAHs like phenanthrene, fluoranthene and fluorine (Sack et al., 1997; Cerniglia and Sutherland, 2010) (and the references therein).

### Catabolism of PAHs by Non-ligninolytic Fungi

Polycyclic aromatic hydrocarbons metabolic pathway of non-ligninolytic fungi is similar to those formed by mammalian enzymes. In this case, the predominant pathway of initial oxidation of PAHs by non-ligninolytic fungi involves the activity of the cytochrome P450 monooxygenase enzymes. These enzymes catalyze a ring epoxidation to form an unstable arene oxide, which is further transformed to *trans*-dihydrodiol *via* an epoxide-hydrolase catalyzed reaction (Jerina, 1983; Sutherland et al., 1995). Non-ligninolytic fungi such as *Cunninghamella elegans* and ligninolytic fungi such as *Pleurotus ostreatus*, metabolize PAHs *via* this pathway (Bezalel et al., 1996; Tortella et al., 2005). For example, the transformation of fluoranthene by *C. elegans* produce fluoranthene trans-2,3-dihydrodiol, 8- and 9-hydroxyfluoranthene trans-2,3-dihydrodiols (Tortella et al., 2005). Similarly, *P. ostreatus* metabolizes pyrene into pyrene trans-4,5-dihydrodiol (Bezalel et al., 1996). Arene oxide produced by cytochrome P450 can also be rearranged to phenol derivatives by non-enzymatic reactions and are subsequently conjugated with sulfate, xylose, glucoronic acid, or glucose (Mueller et al., 1996; Pothuluri et al., 1996). In some fungi, cytochrome P450 monooxygenases oxidize PAHs to epoxides and dihydrodiols which are potent carcinogens and more toxic than the respective parent PAHs, while on the other hand peroxidase-mediated oxidation of PAHs produces quinines which are less toxic than the parent PAHs (Cerniglia and Sutherland, 2010). This is why oxidation of PAHs by ligninolytic enzymes could be a more logical strategy for the detoxification of PAHs contaminated environment.

### Catabolism of PAHs by Ligninolytic Fungi

**FIGURE 7:**
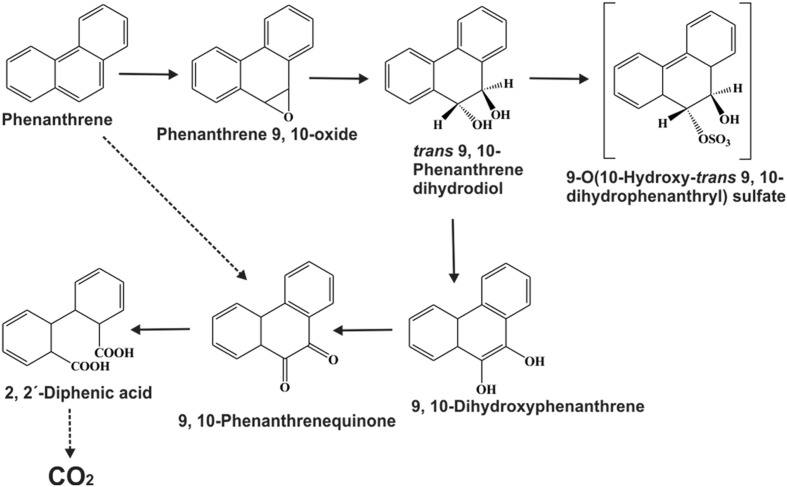
**Proposed phenanthrene degradation pathway by the ligninolytic fungus *Pleurotus ostreatus* (adapted from Bezalel et al., 1997)**.

White-rot fungi are ubiquitous in nature and can produce ligninolytic enzymes which are secreted extracellularly. These enzymes can degrade lignin present in wood and other organic substances. Ligninolytic enzymes are mainly of two types, peroxidases and laccases. On the basis of reducing substrate types, peroxidase enzyme can be classified again into two types, lignin peroxidase and manganese peroxidase. Both types of peroxidases have the ability to oxidize PAHs (Hammel, 1995; Cerniglia and Sutherland, 2010). Laccases, the phenol oxidase enzymes, also have the ability to oxidize PAHs (Cerniglia and Sutherland, 2010). Compared to bacterial PAHs degrading enzymes, ligninolytic enzymes are not induced in the presence of PAHs or by its degradation products (Verdin et al., 2004). As ligninolytic enzymes are secreted extracellularly, they are able to diffuse toward the immobile PAHs, and that is why they are more useful than bacterial intracellular enzymes in making initial attack on PAHs in soil. In addition compared to bacterial PAH-degrading enzymes, ligninolytic enzymes have broad substrate specificity and are therefore able to transform a wide range of substrates, even those which are most recalcitrant (Tortella et al., 2005; Cerniglia and Sutherland, 2010). Ligninolytic enzymes can transform PAHs by producing hydroxyl free radicals by the donation of one electron, which oxidizes the PAH ring (Sutherland et al., 1995). As a result, PAH-quinones and acids are formed instead of dihydrodiols. It has been seen that ligninolytic fungi mineralize PAHs by a combination of ligninolytic enzymes, cytochrome P450 monooxygenases, and epoxide hydrolases (Bezalel et al., 1997). Numerous reports have been documented on the degradation of PAHs by white-rot fungi (Tortella et al., 2005; Cerniglia and Sutherland, 2010). The metabolic pathway for the degradation of phenanthrene by the ligninolytic fungus *Pleurotus ostreatus* is illustrated in **Figure 7** (Bezalel et al., 1997). PAHs degradative potential of wood-rotting fungi *Plrurotus ostreatus* and *Antrodia vaillantii* was also examined in soil, artificially contaminated with fluorene, phenanthrene, pyrene, and benz[a]anthracene (Andersson et al., 2003). It has been reported that although *P. ostreatus* significantly increased the degradation of PAHs in soil, but in the process, accumulated toxic PAH metabolites. It has also been seen that this white-rot fungus inhibits the indigenous microbial populations in the soil, which may have prohibited the complete mineralization of the PAHs. Conversely, the brown-rot fungus *A. vaillantii* did not generate any dead-end metabolites, although its PAHs degradation rate was similar to that of *P. ostreatus* (Andersson et al., 2003). Oxidation of pyrene, anthracene, fluorene and BaP to the corresponding quinines by lignin peroxidase and manganese peroxidase was reported by the white-rot fungus *Phanerochaete chrysosporium* (Bogan et al., 1996a,b). In another study, complete mineralization of BaP (HMW PAHs) by the white-rot fungus *P. chrysosporium* was reported in a two-stage pilot scale reactor (May et al., 1997). Clemente et al. (2001) studied PAHs degradation by thirteen ligninolytic fungal strains and reported that the rate of degradation varies with a variation of lignolytic enzymes (Clemente et al., 2001). In this study, highest naphthalene degradation (69%) was reported by the strain 984 which have Mn-peroxidase activity, followed by strain 870 (17%) having lignin peroxidase and laccase activities. The ability of soil fungi to degrade PAHs that produce ligninolytic enzymes was also studied under microaerobic and very-low-oxygen conditions (Silva et al., 2009). It was reported that *Aspergillus* sp., *Trichocladium canadense*, and *Fusarium oxysporum* can degrade LMW PAHs (2–3 ring compounds) most extensively, whereas highest degradation of HMW PAHs (4–7 rings) was observed in *T. canadense*, *Aspergillus* sp., *Verticillium* sp., and *Achremonium* sp. These results suggest that, along with bacteria, fungi can be exploited as a valuable endeavor for the bioremediation of PAH contaminated sites.

## Microalgal Degradation of PAHs

**FIGURE 8:**
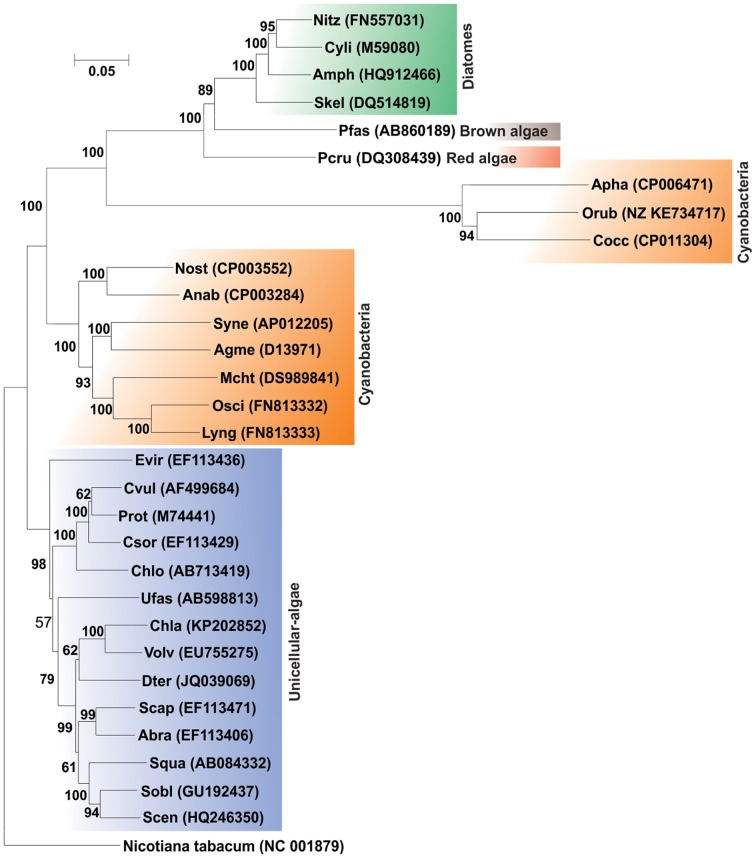
**Phylogenetic tree based on ribulose 1, 5-bisphosphate carboxylase (*rubisco*) gene sequence (large subunit) of organisms enlisted in Supplementary Table S2**. Organism designations are according to those given in Supplementary Table S2. *rubisco* gene sequences are taken from representative strains present in NCBI and the respective accession numbers are given within parentheses. rubisco gene sequence (large subunit) of organism *Nicotiana tabacum* was used as outgroup. Numbers at nodes indicate levels of bootstrap support based on neighbor-joining analysis of 100 resampled datasets. Bootstrap values below 60% are not shown. Bar represents 0.05 substitutions per nucleotide position.

**FIGURE 9:**
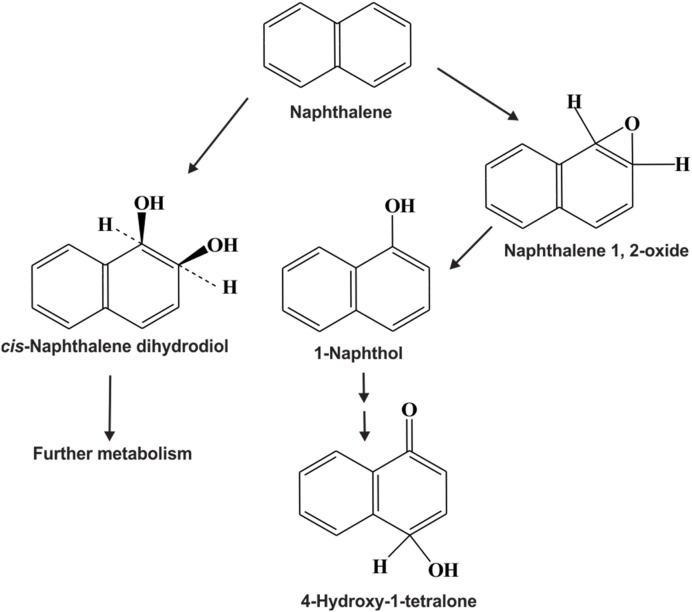
**Proposed naphthalene transformation pathway by the cyanobacteria, *Oscillatoria* sp. strain JCM (adapted from Cerniglia et al., 1980a)**.

Compared to bacteria and fungi, relatively little attention has been paid to the biodegradation of PAHs by microalgae (cyanobacteria, diatoms etc.). Microalgae are one of the major primary producers in aquatic ecosystems, and play vital roles in the fate of PAHs in those environments. Several strains of microalgae are known to metabolize/transform naphthalene, phenanthrene, anthracene, BaP and other PAHs (Cerniglia et al., 1979, 1980a,b; Narro et al., 1992a,b; Safonova et al., 2005; Chan et al., 2006; El-Sheekh et al., 2012). Supplementary Table S2 represents different microalgal strains involved in the bioremediation of various PAHs. **Figure 8** illustrates the *rubisco* large subunit gene-based phylogenetic analysis of all the organisms enlisted in Supplementary Table S2. **Figure 9** shows the biotransformation pathway of naphthalene by microalgae *Oscillatoria* sp., strain JCM (Cerniglia et al., 1980a). Under photoautotrophic growth conditions, strain JCM has been reported to oxidize naphthalene to 1-naphthol, whereas marine cyanobacterium *Agmenellum quadruplicatum* strain PR-6 can convert phenanthrene to phenanthrene trans-9,10-dihydrodiol and 1-methoxyphenanthrene (Narro et al., 1992a,b). In a separate study, oxidation of BaP by the microalgae *Selanastum capricornutum* has been evaluated. Transformation of BaP resulted in the formation of cis-4,5-, 7,8-, 9,10- and 11,12-BaP-dihydrodiols, involving a dioxygenase system, similar to bacterial PAH degradation systems but unlike those of eukaryotic organisms (like fungi) which involve monooxygenase systems (Warshawsky et al., 1988). In another study, the effects of gold, white or UV-A fluorescent lights for the biotransformation of BaP and phototoxicity of carcinogenic PAHs in different algal systems was determined. It has been found that algae like *S. capricornutum, Scenedesmus acutus* and *Ankistrodesmus braunii* are able to degrade BaP to dihydrodiols, and the degradation varies with the kind and intensity of light sources (Warshawsky et al., 1995). The removal efficiency for either fluoranthene or pyrene, or a mixture of fluoranthene and pyrene were also determined using *Chlorella vulgaris*, *Scenedesmus platydiscus*, *Scenedesmus quadricauda*, and *Selenastrum capricornutum* microalgal species (Lei et al., 2007). After 7 days of treatment PAHs removal by *S. capricornutum* and *C. vulgaris* was 78 and 48% respectively. The removal rate of fluoranthene and pyrene in a mixture was found to be similar, or higher than the respective single compound, which indicates that the presence of one PAH acts synergistically in the removal of the other PAH (Lei et al., 2007). In another study, microalgal strain *Prototheca zopfii* immobilized in polyurethane foam has been reported to accumulate a mixture of PAHs in the matrix, when incubated with mixture of aliphatic hydrocarbons and PAHs as substrates. Though the immobilized organism can degrade n-alkanes but it’s PAH degradation rate is very less, however, PAHs accumulation did not impair the degradation of PAHs; whereas in case of free-living cells, the organisms can reduce the concentration of both PAHs and *n*-alkanes satisfactorily (Ueno et al., 2006, 2008). Hong et al. (2008) have examined the accumulation and biodegradation of phenanthrene (Phn) and fluoranthene (Fla) by the two diatoms *Skeletonema costatum* and *Nitzschia* sp., enriched from a mangrove aquatic ecosystem. It was seen that the strains were capable of accumulating and degrading phenanthrene and fluoranthene simultaneously and the PAHs accumulation and degradation capability of *Nitzschia* sp. were higher than those of *S. costatum*. Further, it had been observed that the degradation of fluoranthene by the two diatoms was slower, compared to phenanthrene. The strains also showed similar or higher efficiency in the removal of the Phn–Fla mixture than Phn or Fla alone (Hong et al., 2008). In another study, the efficiency of seven microalgal species to remove pyrene from solution was reported (Lei et al., 2002). In a recent study removal of benzo(a)pyrene (BaP) by sorption and degradation was determined by two microalgal species *Selenastrum capricornutum* and *Scenedesmus acutus* (Garcia de Llasera et al., 2016). It has been seen that *S. capricornutum* can remove 99% of BaP after 15 h of exposure, whereas *S. acutus* can remove 95% after 72 h of exposure. In a separate study, the effects of metals on biosorption and biodegradation of fluorene, phenanthrene, fluoranthrene, pyrene and benzo[a]pyrene by *Selenastrum capricornutum* were investigated (Ke et al., 2010). It has been shown that both metal dosage and exposure time yielded a significant effect on the ability of removal of low molecular weight PAHs like fluorene and phenanthrene by the alga, whereas for high molecular weight PAHs like fluoranthrene, pyrene and BaP, the removal efficiency was not affected by the presence of metals. Patel et al. (2016) recently reported the biodegradation of anthracene and pyrene by *Anabaena fertilissima* (Patel et al., 2016), while Takáčová et al. (2014) reported the biodegradation of BaP by the microalgae *Chlorella kessleri*. Removal and transformation of seven high molecular weight PAHs in water was reported by live and dead cells of a freshwater microalga, *Selenastrum capricornutum* under gold and white light irradiation. The removal efficiency of PAHs, and the effectiveness of live and dead cells, was found to be predominantly PAH dependent (Ke et al., 2010; Luo L. et al., 2014; Luo S. et al., 2014). The first study on the potential of algal–bacterial microcosms was reported for the biodegradation of aromatic pollutants comprising salicylate, phenol and phenanthrene in a one-stage treatment (Borde et al., 2003). The green alga *Chlorella sorokiniana* was grown with those three aromatics at different concentrations, showing increasing inhibitory effects in the order salicylate < phenol < phenanthrene. However, a satisfactory removal (>85%) was achieved only in the system having both bacteria and algae, incubated under continuous lighting, indicating the synergistic relationship between the algal–bacterial microcosms in the removal of organic pollutants. An algal–bacterial consortium consisting of *Chlorella sorokiniana* and *Pseudomonas migulae* was also reported for the degradation of phenanthrene under photosynthetic conditions and without an external source of oxygen (Munoz et al., 2003). In another study, accelerated pyrene degradation by a bacterial-algal consortium was reported under photosynthetic condition (Luo L. et al., 2014; Luo S. et al., 2014). These studies indicate that microalgae can be used singly or along with bacteria as a potential candidate for biodegradation of PAHs. However, more research is needed for optimizing their efficiency to apply them successfully in the bioremediation of PAH-contaminated environment.

## Degradation of PAHs by Microbial Consortia and Co-Metabolism

Occasionally it has been observed that a particular microorganism does not have all the genes required for the complete mineralization of a particular organic pollutant like PAH. Therefore, researchers are developing microbial consortia for complete degradation of such pollutants. In those consortia, each microorganism has specialized role in certain degradation steps where, intermediates produced by certain microorganisms are utilized by other members. On the other hand, cometabolism is a phenomenon by which a recalcitrant compound is degraded in the presence of an analogous degradable compound. Several microorganisms can co-metabolize PAHs, and it is a very complex phenomenon. As PAHs in the environment are present as a mixture, co-metabolism plays a very crucial role for bioremediation of PAHs contaminated sites. Co-metabolism of one PAH could have a synergistic effect on the degradation of other PAHs, specifically for the degradation of HMW PAHs (van Herwijnen et al., 2003). In a report, *Rhodococcus* sp. strain S1 when grown on anthracene has shown to cometabolize phenanthrene to phenanthrene trans-9,10-dihydrodiol (Tongpim and Pickard, 1999). The cometabolic degradation of phenanthrene, fluoranthene, anthracene and dibenzothiophene was reported by the fluorene grown *Sphingomonas* sp. LB126 (van Herwijnen et al., 2003). In a separate study, cometabolic degradation of a creosote-PAHs mixture including phenanthrene, fluoranthene, and pyrene, by the pyrene-degrading strain *Mycobacterium* sp. AP1 was reported (Lopez et al., 2008). However, it has been observed that the rate of degradation of individual PAHs was related to their aqueous solubility, for example, the biodegradation rate of fluoranthene and pyrene are significantly lower than that of phenanthrene (Lopez et al., 2008). In another study, cometabolism of acenaphthene and acenaphthylene by the succinate grown *Beijerinckia* sp. and one of its mutant strain, *Beijerinckia* sp. strain B8/36 was reported (Schocken and Gibson, 1984). Both the wild type and the mutant strains cometabolize acenaphthene to 1-acenaphthenol, 1-acenaphthenone, 1,2-acenaphthenediol, acenaphthenequinone, and 1,2-dihydroxyacenaphthylene. Furthermore, *Sphingobium* sp. strain PNB was observed to co-metabolize fluoranthene, acenaphthene, benz[a]anthracene, pyrene and benzo[a]pyrene, in presence of phenanthrene indicating metabolic robustness of the strain (Roy et al., 2013).

Various white-rot fungi can metabolize PAHs along with bacteria in consortium by improving bioavailability of target compounds. Due to lack of suitable enzymes, generally fungi cannot degrade HMW PAHs completely, but can transform them into comparatively polar metabolite(s) with their extracellular enzymes, which can further be degraded by bacteria and other microbes (Sutherland, 1992). Meulenberg et al. (1997) reported that the degradation product of anthracene by white-rot fungi can be mineralized by indigenous mixed bacterial cultures (e.g., activated sludge or soil) more quickly than anthracene itself (Meulenberg et al., 1997). It has been seen that, inoculation of fungal–bacterial co-cultures into the soils contaminated with PAHs, can significantly enhance degradation of HMW PAHs, like chrysene, benzo-[a]anthracene and dibenzo[a,h]anthracene (Boonchan et al., 2000). Consequently, it is assumed that PAH degradation in nature is a result of coordinate steps mediated by fungi and bacteria, with the fungi playing the initial oxidation step (Meulenberg et al., 1997; Boonchan et al., 2000; Cerniglia and Sutherland, 2010). Along with bacteria and fungi, various microalgal strains have been used for the degradation of PAHs in a consortium. Microalgae can be exploited as a potential candidate for degradation of PAHs specifically in aquatic environments. Metabolic competition is another feature of biodegradation when a combinations of two individually degradable PAHs are present in the medium (Bouchez et al., 1995). For example, when LMW PAHs such as phenanthrene and fluorene present in the medium, they can inhibit the degradation of fluoranthene and pyrene (Dean-Ross et al., 2002). Nevertheless, another cause of inhibition is due to the formation of dead-end products that result from co-metabolic degradation of non-growth substrates (Stringfellow and Aitken, 1995).

## Factors Affecting the Bioremediation of PAHs

**FIGURE 10:**
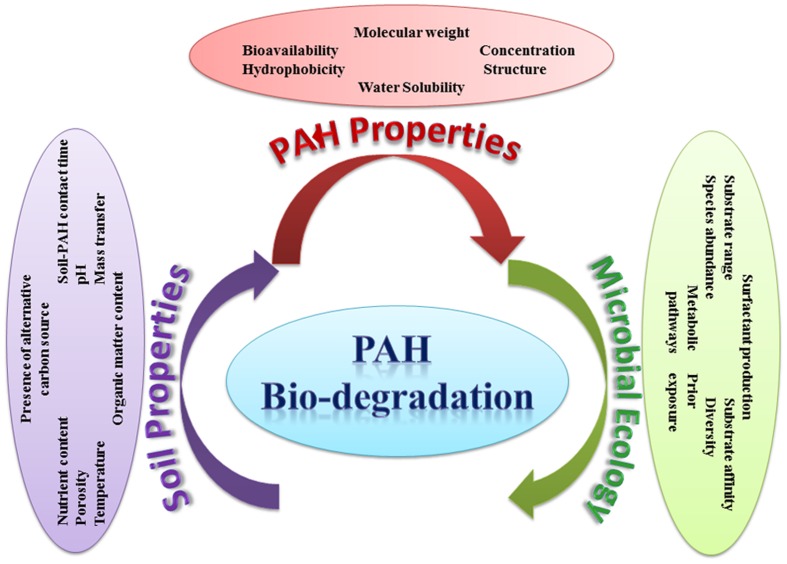
**Abiotic and biotic factors influencing the degradation of PAHs in soil**.

The effectiveness of bioremediation has been mainly investigated under ideal laboratory conditions, having a circum-neutral pH and ambient mesophilic temperature. However, in the real situation, bioremediation can be effective only at sites where environmental conditions permit microbial growth and express associated enzyme activity so that microorganisms can enzymatically attack the pollutants converting them to harmless products. Numerous abiotic and biotic factors (such as pH, nutrient availability and the bioavailability of the pollutants) can apparently differ from site to site, which in turn can influence the process of bioremediation in those environments either by inhibiting or accelerating the growth of the pollutant-degrading microorganisms. **Figure 10** illustrates the various abiotic and biotic factors influencing PAHs degradation in soil. The main environmental factors that could affect the rate of biodegradation of PAHs in the environment are summarized below.

### Temperature

Temperature has a profound effect on the biodegradation of PAHs in contaminated sites since those places are not always at ambient temperature for activity of the inhabitant microorganisms. When temperature increases, the solubility of PAHs also increases, which in turn increases the bioavailability of PAH molecules. On the other hand, with increasing temperature, dissolved oxygen level decreases, as a result of which the metabolic activity of aerobic mesophilic microorganisms reduces. There are some microorganisms called psychrophiles, which can operate at low temperature, and are in contrast to thermophiles that function at high temperature. At high temperature, some pollutants can get transformed into a new compound and often the daughter compound appears to be more toxic than the parent compound, which in turn inhibits its biodegradation rate (Müller et al., 1998). Generally in most of the studies biodegradation of PAHs have been examined under moderate temperatures, however, there are reports on PAHs biodegradation under extreme temperatures. For example, biodegradation of petroleum hydrocarbons including PAHs was reported in seawater at low temperatures (0–5°C) (Brakstad and Bonaunet, 2006) while the biodegradation of PAHs along with long chain alkanes was documented at very high temperature (60–70°C) by *Thermus* and *Bacillus* spp (Feitkenhauer et al., 2003).

### pH

pH also plays a significant role in biodegradation processes including that of PAHs. Usually, microorganisms are pH-sensitive and near neutral conditions (6.5–7.5) are favored by most of them for their normal activity. However, it has been seen that in many PAHs contaminated sites, pH is very far from neutral settings. For example, some abandoned gasworks sites frequently contain a huge amount of demolition waste like concrete and brick. Leaching of these materials can result in the increase of pH of the native soil. Moreover, the leaching of some material like coal spoil can generate an acidic environment due to the oxidation of sulfides. These acidic or alkaline conditions can create adverse conditions for microbial activities, which in turn decrease the biodegradation of PAHs in those sites. Hence, it is recommended to adjust the pH at those sites by adding chemicals: basic soils can be treated with ammonium sulfate or ammonium nitrate while acidic soils can be treated by liming with calcium or magnesium carbonate (Bowlen and Kosson, 1995) to generate an environment for effective biodegradation.

### Oxygen

Biodegradation of organic contaminants including PAHs can proceed under both aerobic and anaerobic conditions; however, most of the studies focus bioremediation of PAHs under aerobic condition, where oxygen acts as a co-substrate and a rate limiting factor. During aerobic biodegradation of PAHs, oxygen is required for the action of both mono- and dioxygenase enzymes in the initial oxidation of the aromatic rings. For *in situ* bioremediation of contaminants, sometimes oxygen is added from an external source. The addition of oxygen externally may be achieved by drainage, tilling, and addition of chemicals that release oxygen or by injection of air into the contaminated site (Pardieck et al., 1992; Atlas and Unterman, 1999; Bewley and Webb, 2001). In a study, enhanced intrinsic bioremediation of BTEX compounds in aquifer using an oxygen releasing compound (magnesium peroxide; ORC^®^) was reported (Odencrantz et al., 1996). Again, in a separate study, *in situ* bioremediation of an aquifer contaminated with various pollutants, including phenols, BTEX compounds and PAHs was reported, in which sodium nitrate was circulated through the aquifer as the oxygen source, *via* a series of injection and abstraction wells (Bewley and Webb, 2001).

### Nutrients

Availability of nutrients is another rate-limiting factor for successful bioremediation of PAHs contaminated environments. Along with easily metabolized carbon source, microorganisms require various minerals like nitrogen, phosphorus, potassium, and iron for normal metabolism and growth. Thus, supplementation of nutrients, in the poor nutrient content pollutant contaminated sites is required to stimulate the growth of autochthonous microorganisms, to enhance bioremediation of pollutants (Atagana et al., 2003). In marine environments, poor biodegradation of petroleum hydrocarbon is due to a low level of nitrogen and phosphorous (Floodgate, 1984). On the other hand, excessive nutrient availability can also inhibit the bioremediation of pollutants (Chaillan et al., 2006). There are several reports on the negative effects of high nutrient levels on the biodegradation of organic pollutants especially PAHs (Carmichael and Pfaender, 1997; Chaîneau et al., 2005).

### Bioavailability

In case of a biological system, the term bioavailability is defined as the fraction of a chemical that can be taken up or transformed by living organisms during the course of the experiment. This fraction can vary under the influence of mass transfer parameters, which include physicochemical processes governing dissolution, desorption and diffusion, hydrological processes like mixing and, finally, biological processes, such as uptake and metabolism (Bosma et al., 1997; Semple et al., 2003). Bioavailability is regarded as one of the most crucial factors in bioremediation of pollutants. Biodegradation of PAHs in the environment is often limited, due to their low aqueous solubility and their strong tendency to sorbs to mineral surfaces (like clays) and organic matters (like black carbon, coal tar humic acids) in the soil matrix, processes which reduce bioavailability. There are numerous reports on poor or unsuccessful remediation of PAHs contaminated sites due to absorption of PAHs in black carbon, coal-tar substances which decreases bioavailability of PAHs (Volkering et al., 1992; Luthy et al., 1993, 1994; Nelson et al., 1996; Northcott and Jones, 2001; Hong et al., 2003; Bucheli et al., 2004; Cornelissen et al., 2005; Liu et al., 2009; Benhabib et al., 2010; Rein et al., 2016). Again, the aqueous solubility of PAHs decreases with increasing molecular weight, which in turn reduces the bioavailability of PAHs. It has been seen that longer any hydrophobic organic contaminants (like PAHs) is in contact with soil, the more irreversible the sorption, and lesser is the chemical and biological extractability of the contaminants, which is known as ‘aging’ of the contaminant (Alexander, 2000; Luo et al., 2012). It has been reported that extractability and bioavailability of PAHs decrease significantly with time in aging processes which can be significantly rate-limiting for *in situ* bioremediation (Alexander, 2000; Megharaj et al., 2002; Luo et al., 2012; Abdel-Shafy and Mansour, 2016).

### Inhibition Due to Excess Substrate or End Product

The major goal of bioremediation is the removal or detoxification of contaminants from a given environment. However, sometimes it is possible that the contaminant gets transformed into a more toxic dead end product. This is why it is essential to confirm that the pollutant is completely mineralized at the end of the treatment (Mendonca and Picado, 2002; Lundstedt et al., 2003). A study using a bioreactor to treat PAH-contaminated gasworks soil using *in situ* bioremediation monitored both the removal of PAHs and the accumulation of oxy-PAHs, such as PAH-ketones, quinines and coumarins as dead end products (Lundstedt et al., 2003). As oxy-PAHs are more toxic than the parent PAHs, this study highlights the significance of monitoring the metabolites during bioremediation, specifically for toxic dead-end products, and determining the toxicity of the metabolites both before and after treatment (Lundstedt et al., 2003).

Since PAHs in the environment are present as mixtures, the effect of substrate interaction during biodegradation is crucial in determining the fate of PAHs in nature. It has been reported that, when present as a mixture, the high molecular weight PAHs are degraded followed by the degradation of low molecular weight PAHs, by the bacterial community (Mueller et al., 1989). It has been also observed that a high concentration of naphthalene have inhibitory effect on the degradation of other PAHs, by a defined bacterial coculture (Bouchez et al., 1995). Similarly, a competitive inhibition of phenanthrene degradation by naphthalene, methylnaphthalene and fluorene in binary mixtures using two pure cultures was reported (Stringfellow and Aitken, 1995). Thus during *in situ* bioremediation of PAHs, concentration of PAHs in the contaminated sites, and a chance for the formation of the toxic dead end product(s) need to be considered for effective process development.

## Recent Advancement in Molecular Techniques in Understanding Bacterial Degradation of PAHs and Future Directions

Nowadays, bioremediation has become an intensive area of research. However, more advancement has to be made in developing practically efficient microbial bioremediation processes. Although microorganisms have the capability to use numerous organic pollutants as their carbon and energy sources, their proficiency at eliminating such pollutants might not be of top class level for cleaning up present-day pollution. In fact, microorganisms have evolved in the direction of ecological fitness rather than biotechnological efficacy (Schmid et al., 2001). To enhance the catabolic efficiency of a microorganism for bioremediation, bioengineering is a prerequisite. Recent advancement in genetic, genomic, proteomic and metabolomic technologies, which are applied to study bioremediation of organic pollutants, have contributed considerably to enrich our knowledge on various aspects of the physiology, ecology, biochemistry and regulatory mechanisms of the microbial catabolic pathways. Hence, practical utilization of that knowledge is essential to manipulate or reconstruct as well as enhance the natural processes, thereby making strategies for the development of more competent biocatalysts for various biotechnological applications including bioremediation of culprit organic pollutants in the contaminated sites and transformation of toxic chemical into harmless product or other specialty chemicals (Schmid et al., 2001; Carmona et al., 2009).

A considerable advancement in microbial ecology was achieved based on the identification of conserved sequences present in a particular group of microorganisms, most notably the 16S rRNA or 18S rRNA genes which could provide a phylogenetic characterization of the microbial population present in a particular habitat (Pace et al., 1986; Amann et al., 1995). This approach is a significant achievement in the field of bioremediation because by determining the microbiota in a polluted environment it is possible to identify particular microorganisms inhabiting those environments, thereby predicting possible bioremediation potential. With the advent of Denaturing Gradient Gel Electrophoresis (DGGE), it is now possible to analyze the microbial community structure and dynamics of a particular habitat, more precisely (Gallego et al., 2014; Vila et al., 2015). Development of fluorescence *in situ* hybridization (FISH), which is another technique is quite useful in this field and has often been practiced (Amann et al., 2001).

Generally, there is a positive link between the relative abundance of the genes associated with pollutant removal and the efficiency of bioremediation. However, sometimes it is possible that the genes associated with pollutant removal can be present but not expressed. So, there is an increasing interest in quantifying the mRNA for key catabolic genes *via* real-time PCR (Schneegurt and Kulpa, 1998; Debruyn and Sayler, 2009). In addition transcriptomics, DNA-based stable isotope probing, single cell genomics and DNA microarray techniques are also emerging for application in the field of bioremediation (Wilson et al., 1999; Denef et al., 2005, 2006; Chain et al., 2006; Parnell et al., 2006; Macaulay and Voet, 2014; Mason et al., 2014; Mishamandani et al., 2014). DNA microarray which is a high-throughput version of DNA hybridization technique can detect an enormous number of genes in a single test. One of the recent applications of microarray in the PAHs biodegradation is the construction of GeoChip (He et al., 2010; Nostrand et al., 2012).

The advancement in genome sequencing technology practically revolutionized the field of bioremediation. By whole genome sequencing, it is now possible to study the physiology of microorganisms associated with pollutant removal in more details (Nierman and Nelson, 2002; Buermans and den Dunnen, 2014). Presently many complete or nearly complete genome sequences of cultivable microorganisms are available which have potential catabolic activity. However, unfortunately by using modern microbiological and molecular techniques over the past 40 years so far only an extremely small fraction (~1%) of the total microbial diversity has been cultivated from all habitats investigated (Hugenholtz and Pace, 1996; Hugenholtz et al., 1998). While culturing novel environmental microorganisms is indispensable (Zinder, 2002), perhaps the greatest promise to date comes from metagenomic approaches, which are the only ones currently available to adequately utilize the tremendous microbial diversity- one of the richest sources on earth (Handelsman et al., 1998). Although there are some associated challenges in metagenome-based approaches, scientists already overcame many of those problems (Liles et al., 2008; Mareckova et al., 2008). The progressively decreasing cost and increasing speed of DNA sequencing has made it possible to sequence billions of bases of metagenomic DNA. Culturable aromatics degrading bacteria are being isolated mostly on the basis of their ability to utilize aromatic compound as their sole carbon and energy sources and by genome sequencing, scientist can identify all the genes necessary to completely degrade the compound. Therefore, inclusive knowledge of the catabolic potential of a contaminated site virtually remains unknown by using culture-dependent techniques. High throughput metagenomics reduces that bottleneck. However, at present, relatively little resources have been spent for the sequencing of soil or marine metagenomes contaminated with aromatic pollutants, compared to those committed to the human microbiome (Blow, 2008). That is why there is a scarcity of information about the xenobiotics degrading novel bacteria that are difficult to culture in the laboratory. Sites contaminated with toxic chemicals have become biotechnological gold mines because the indigenous microorganisms may have evolved the necessary enzymes, to degrade or transform those toxic contaminants to an inanimate one, quite different from those found in common cultivable microorganisms (Handelsman et al., 1998; Galvao et al., 2005; Boubakri et al., 2006). In addition, sequencing of the soil or aquatic metagenome will also provide insights into the ecology of microorganisms which in turn will help to identify who the dominant and rare community members are and what are their probable roles in the degradation of recalcitrant molecules like PAHs and other related xenobiotics.

**FIGURE 11:**
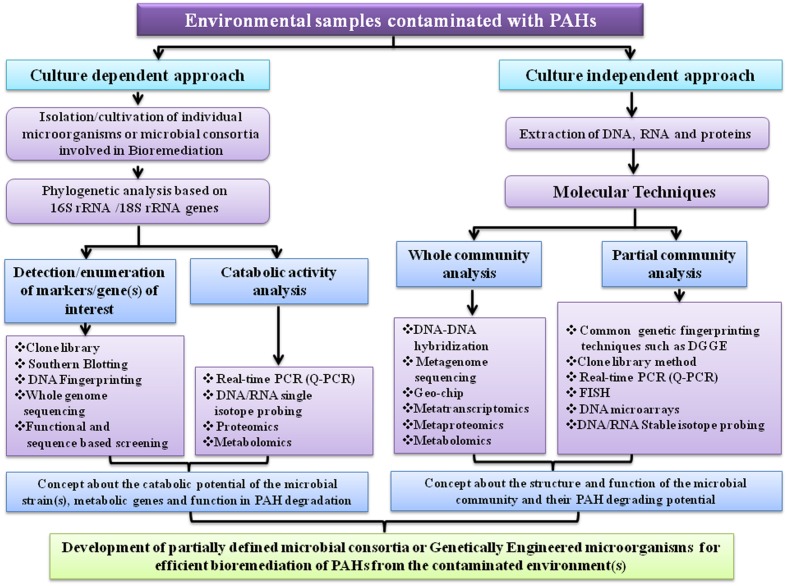
**Schematic diagram summarizing different molecular techniques as future directions for better understanding of the microbial PAH degradation and ecologically sustainable bioremediation of sites contaminated with PAHs**.

Recently another two techniques metaproteomics and metabolomics have been utilized to unfold various aspects of environmental microbiology and have shown their promise in the field of bioremediation (Nesatyy and Suter, 2007; Keum et al., 2008). Proteomics is an efficient technology to recognize proteins and their roles associated with catabolism of PAHs while metabolomics can be exploited to identify metabolites produced during PAHs biodegradation. **Figure 11** illustrates a summarized representation of the molecular techniques involved in studying microbial degradation of PAH. In near future, functional metagenomics, metaproteomics, metabolomics, metatranscriptomics and DNA microarrays will become crucial tools to elucidate the mechanisms of biodegradation of PAHs in the environment, and will offer more information on as-yet-uncultured organisms associated with PAHs bioremediation (Wilson et al., 1999; Amann et al., 2001; Fromin et al., 2002; Liang, 2002; Zhou and Thompson, 2002; Baldwin et al., 2003; Ginige et al., 2004). Moreover, *in silico* biology is being progressively applied in different aspects in the field of bioremediation (Kweon et al., 2010; Chakraborty et al., 2012; Khara et al., 2014). Thus, it is expected that emerging molecular biological, analytical and computational methods which can predict the activity of microorganisms involved in biodegradation, should transform the bioremediation field from a largely empirical practice into a branch of modern science.

## Approaches to the Bioremediation of PAH-Contaminated Environments

Various types of bioremediation technologies can be employed for contaminant removal. Intrinsic bioremediation (also called natural attenuation or ‘*in situ*’ bioremediation) depends on the bioremediation potential of the indigenous micro flora dwelling at the site contaminated with pollutants. These techniques provide the treatment at the site itself avoiding excavation and transport of contaminants, which makes them the most desirable options due to lower cost and fewer disturbances. On the other hand extrinsic bioremediation (also called ‘*ex-situ*’ bioremediation) mainly involves the physical removal of the contaminated material to another location for treatment (Carberry and Wik, 2001). While in the case of “biostimulation” process, it is generally possible to stimulate the indigenous microorganisms to use the contaminants as a food source at a much greater rate by compensating limiting parameters, for example, by introducing additional oxygen or nutrients to the indigenous population (Straube et al., 2003). Another method, “bioaugmentation,” involves introduction of exogenous microorganisms into the contaminated environment which are capable of degrading the target pollutants, either with or without additional nutrients (Straube et al., 2003). Whereas, “humification” is a process by which strongly persistent substances in the polluted environment are incorporated into the humic substances mostly by means of enzymatic reactions (Megharaj et al., 2011). In addition, “phytoremediation” involves removal of environmental contaminants using plants (Haritash and Kaushik, 2009; Megharaj et al., 2011). Another bioremediation technology called ‘landfarming,’ is used to stimulate indigenous biodegradative microorganisms by providing nutrients, water and oxygen, in order to facilitate aerobic degradation of contaminants (Megharaj et al., 2011). While ‘composting’ involves the degradation of pollutant along with other agricultural wastes such as manure etc. (Semple et al., 2001). Hence, it becomes clear that regardless of the method chosen, an ideal bioremediation strategy can be designed only on the basis of knowledge about the microorganisms and the contaminant present within the polluted environments, along with their catabolic potential and response to changes in the environmental conditions.

### Treatment of Soils and Sediments

There are some reports on the treatment of PAH contaminated soils and sediments by *in situ* or *ex situ* bioremediation methods. Straube et al. (2003) reported that a pilot-scale landfarming treatment of PAH-contaminated soil from a wood treatment facility was achieved by biostimulation of the soil with water, ground rice hulls as a bulking agent, and palletized dried blood as a nitrogen source and bioaugmentation of the microbial community with an inoculum of *Pseudomonas aeruginosa* strain 64 (Straube et al., 2003). It has been seen that within 1 year of treatment ~86% of total PAHs were removed from the soil including a moderate reduction in HMW PAHs such as pyrene, BaP etc. In a separate study on the treatment of an aged gasworks soil contaminated with aromatic compounds including PAHs, it has been observed that LMW PAHs and heterocyclic compounds were degraded more quickly than the HMW counterparts (Lundstedt et al., 2003). In addition, the unsubstituted PAHs were degraded faster than the related alkyl-PAHs as well as nitrogen-containing heterocyclics. Treatment of soil at a tar contaminated site *via* composting along with conventional land treatment process revealed that composting led to more extensive PAH removal than did by two different land treatment processes (Guerin, 2000). Sasek et al. (2003) reported the remediation of a manufactured-gas plant soil contaminated with PAHs *via* composting. In this study, treatment of soil was performed in a thermally insulated chamber using mushroom compost containing wheat straw, chicken manure and gypsum, where at the end of 54 days, removal of ~20–60% of individual PAHs was reported, while ~37–80% of individual PAHs degradation was observed after another 100 days of composting (Sasek et al., 2003). Moreover, studies on the removal of PAHs present in contaminated soil using the associated bacterial communities in two aerobic, lab-scale, slurry-phase bioreactors, which were run semi-continuously and fed either on weekly or monthly basis, showed that most of the PAHs, including HMW PAHs were biodegraded to a greater extent in the weekly fed bioreactor for up to 76% (Singleton et al., 2011).

### Treatment of Waters

Polycyclic aromatic hydrocarbons-polluted groundwater can also be bioremediated through both *in situ* and *ex situ* bioremediation procedures. However, due to the high cost associated with the extraction and shipment, *ex situ* treatment of contaminated groundwater is not generally performed. Few reports are documented on the treatment of PAH contaminated water. Bewley and Webb (2001) reported the *in situ* bioremediation of an aquifer which was contaminated with diverse pollutants, including phenols, BTEX compounds and PAHs. The site was bioremediated using a procedure involving a combination of bioaugmentation and biostimulation. Nutrients (a commercial mixture of urea and diammonium phosphate), a commercially available phenol-degrading mixed bacterial inoculum (PHENOBAC, Microbac Ltd, Durham), and sodium nitrate (an oxygen source) were circulated through the aquifer *via* a series of injection and abstraction wells. After treatment for over two and half years, the mean concentration of PAHs was reduced to 0.9 μgL^-1^ from 11 μgL^-1^ along with reduction of other contaminant (Bewley and Webb, 2001). Apart from that, groundwater remediation technology of petroleum-derived compounds (PDCs) including PAHs based on enhanced solubility of PDCs in humic acid was also reported (Van Stempvoort et al., 2002). Zein et al. (2006) reported the treatment of groundwater contaminated with PAHs, gasoline hydrocarbons, and methyl tert-butyl ether using an *ex situ* aerobic biotreatment system and it was observed that after 10 months of treatment, the concentration of PAHs was reduced to >99% along with other pollutants (Zein et al., 2006).

### Treatment with Genetically Engineered Microorganisms (GEMs)

The bioremediation of PAHs contaminated site is generally very slow because there are a number of biotic and abiotic factors responsible for successful bioremediation. In addition, the incomplete success rates might be due to the fact that some places are heavily contaminated, and hence, the microorganisms are incapable to grow and degrade the contaminant at the same rate at which they are introduced into the environment. Although there is very few information on the use of GEMs in bioremediation, they can be a promising candidate for such processes (Ang et al., 2005; Singh et al., 2008; Megharaj et al., 2011). Using genetic engineering it is possible to enhance the activity or broad substrate specificity of certain enzymes associated with PAH-degrading pathways, which in turn will improve the mineralization of those pollutants in the environment (Timmis et al., 1994; Timmis and Pieper, 1999; Ang et al., 2005; Singh et al., 2008). In recent times, various molecular biology tools such as gene conversion, gene duplication, and transposon or plasmids mediated gene delivery are available, which might play vital roles to boost up the biodegrading potential of naturally occurring microorganisms (Timmis et al., 1994; Timmis and Pieper, 1999; Ang et al., 2005; Singh et al., 2008; Megharaj et al., 2011). However, it is crucial to ensure the stability of GEMs prior to their field application since the catabolic activity of released GEM is associated largely with the stability of the recombinant plasmid introduced into the organism (Brunel et al., 1988; Shaw et al., 1992; Samanta et al., 2002). As GEMs that are released into a contaminated site can spread to other places and multiply under favorable conditions, so before releasing into the environment there should be a clear understanding of the possible side effects associated with GEMs, which would possibly guide the restriction of their application in pollutant abatement (Samanta et al., 2002; Ang et al., 2005; Singh et al., 2008; Megharaj et al., 2011).

## Conclusion

In last few decades, there has been a great deal of progress in the study of the bioremediation of PAHs. Numerous microorganisms have been isolated and characterized having PAHs catabolic potentials. In addition, many unique enzymes with different catabolic efficiency associated with PAHs degradation have been purified and different novel biochemical pathways for PAHs degradation have been elucidated. Moreover, many PAH catabolic operons have been sequenced, and their regulatory mechanism for PAH degradation has been determined. The advancement in genetic, genomic, proteomic and metabolomic approaches, which are employed to study catabolism of organic pollutants have contributed remarkably in understanding the physiology, ecology, biochemistry of PAHs degrading microorganisms. However, more detail research is a prerequisite to determine exactly what is going on in PAH-contaminated environment. In addition, there are still various aspects of bioremediation of PAHs that remain unknown or otherwise have insufficient information, which requires future attention. There is very scarce knowledge on genes, enzymes as well as the molecular mechanism of PAHs degradation in high salinity environments, or anaerobic environments. Also, there is very little information on the transmembrane trafficking of PAHs and their metabolites. Various transporters have been assumed to be participating in the transport of PAHs into microorganisms, but till date, none has been characterized.

There are various factors which may affect bioremediation of PAHs in a contaminated environment. Adjustment of oxygen concentration, pH, temperature, nutrient availability and improvement of bioavailability may increase PAHs degradation. It is seen that in contaminated soils and sediments, PAHs get entrapped in coal tar or black carbon particles, which results in unsuccessful remediation due to decrease in PAHs bioavailability (Bucheli et al., 2004). This phenomenon is a major bottleneck for successful remediation of PAHs contaminated environments (Ortega-Calvo and Alexander, 1994). Some microorganisms are known to excrete biosurfactants which enhance the bioavailability of organic pollutants. Many microorganisms exhibit chemotaxis toward pollutants. These strategies lead to enhanced degradation of organic pollutants. The addition of small amount of biosurfactant, which increases the bioavailability of PAHs, or some merely toxic chemicals, like salicylic acid, which induce PAHs catabolic operons may enhance biodegradation of PAHs in the environment. It has been seen that organic amendments influence the indigenous microbial community as well as efficiency of bioremediation of PAHs in contaminated soil (Gandolfi et al., 2010; Loick et al., 2012; Lukić et al., 2016; Rein et al., 2016). So adjustment of organic matter content of a polluted site with exogenous addition of compost or other substances like buffalo manure, food and vegetables waste may enhance the bioremediation efficiency of PAH polluted site. In addition, instead of treating with a single microorganism, a defined microbial consortium may give a better result in some cases for successful remediation of a contaminated site. So, more research is required on the catabolic capabilities of PAHs degrading microbial consortia, which will further deepen our understanding on the microbial consortia-mediated remediation of contaminated environments, and will help to develop potential microbial consortium having robust pollutant degrading potential. It is also possible to treat the contaminated site sequentially with fungi, bacteria and algae or in combination for more efficient pollutant removal.

In addition, genetic engineering can be employed to boost the catabolic efficiency of microorganisms used in bioremediation. Today, scientists are capable to create unique PAHs metabolic pathways by recombining different catabolic genes from different organisms in a single host cell. In this way it is possible to enhance the substrate specificity of a catabolic pathway to degrade new substrates, complete partial pathways and also to construct novel pathways not found in nature, which may permit the mineralization of highly recalcitrant compounds and avoid the accumulation of toxic dead end product. However, before releasing such GMOs into the environment, it is necessary to check thoroughly the possibility of any unwanted side effects produced by those GMOs. In addition, authorities should be convinced that the GMOs are safer, cheaper, and more efficient than the present alternatives. Thus, it is anticipated that the compiled information present in this manuscript will open up new avenues to the researchers, and the field of bioremediation will revolutionize in near future.

## Author Contributions

DG translated the concept and wrote the manuscript. DG and SG analyzed the data, edited and formatted the manuscript. TD and YA generated the concept and ideas, critically revised the manuscript and approved the final version for publication.

## Conflict of Interest Statement

The authors declare that the research was conducted in the absence of any commercial or financial relationships that could be construed as a potential conflict of interest.
