# Corrigendum: Current State of Knowledge in Microbial Degradation of Polycyclic Aromatic Hydrocarbons (PAHs): A Review

**DOI:** 10.3389/fmicb.2016.01837

**Published:** 2016-11-15

**Authors:** Debajyoti Ghosal, Shreya Ghosh, Tapan K. Dutta, Youngho Ahn

**Affiliations:** ^1^Environmental Engineering Laboratory, Department of Civil Engineering, Yeungnam UniversityGyeongsan, South Korea; ^2^Disasters Prevention Research Institute, Yeungnam UniversityGyeongsan, Korea; ^3^Department of Microbiology, Bose InstituteKolkata, India

**Keywords:** biodegradation, Polycyclic Aromatic Hydrocarbons (PAHs), bacteria, fungi, algae

Due to an inadvertent error, Louvado et al. (2015) was noted cited in the footnotes of Table 1. The corrected Table 1 along with the footnote appears below. The authors apologize for this error. This does not affect the scientific conclusions of the article in any way.

**Table 1:** **Physical-chemical properties and some relevant information of 16 PAHs enlisted as priority pollutants by US EPA**.

**Name**	**Molecular formula**	**Cas registry no**.	**Physical chemical properties**				**Toxicology**			**Biodegradation**	
			**B. Pt. (°C)**	**M.Pt (°C)**	**V.P. (mmHg at 25°C)^a^**	**Solubility (mg/L)^a^, ^b^**	**TEF^a^, ^c^**	**IARC^a^, ^d^**	**EPA^a^, ^e^**	**Estimated half-lives (days)^a^, ^f^**	**Measured half-lives (days)^a^, ^g^**
Naphthalene	C_10_H_8_	91-20-3	218	80.2	8.5 × 10^−2^	31	n.d.	2B	C	5.66	n.d.
Acenaphthene	C_12_H_10_	83-32-9	279	93.4	2.5 × 10^−3^	3.93	0.001	3	D	18.77	n.d.
Acenaphthylene	C_12_H_8_	208-96-8	280	91.8	6.68 × 10^−3^	1.93	0.001	n.c.	D	30.7	n.d.
Anthracene	C_14_H_10_	120-12-7	342	216.4	6.53 × 10^−6^	0.076	0.01	3	D	123	2.7
Phenanthrene	C_14_H_10_	85-01-8	340	100.5	1.2 × 10^−4^	1.20	0.001	3	D	14.97	5
Fluorene	C_13_H_10_	86-73-7	295	116-7	6.0 × 10^−4^	1.68–1.98	0.001	3	D	15.14	n.d.
Fluoranthene	C_16_H_10_	206-44-0	375	108.8	9.22 × 10^−6^	0.20–0.26	0.001	3	D	191.4	9.2
Benzo[*a*]anthracene	C_18_H_12_	56-55-3	438	158	4.11 × 10^−3^	0.010	0.1	2B	B2	343.8	>182
Chrysene	C_18_H_12_	218-01-9	448	254	6.23 × 10^−9^	1.5 × 10^−3^	0.010	2B	B2	343.8	n.d.
Pyrene	C_16_H_10_	129-00-0	150.4	393	4.5 × 10^−6^	0.132	0.001	3	D	283.4	151
Benzo[*a*]pyrene	C_20_H_12_	50-32-8	495	179	5.49 × 10^−9^	3.8 × 10^−3^	1.0	1	B2	421.6	11
Benzo[*b*]fluoranthene	C_20_H_12_	205-99-2	481	168.3	5.0 × 10^−7^	0.0012	n.d.	2B	B2	284.7	n.d.
Benzo[*k*]fluoranthene	C_20_H_12_	207-08-9	480	215.7	9.7 × 10^−10^	7.6 × 10^−4^	0.1	2B	B2	284.7	n.d.
Dibenzo[a,h]anthracene	C_22_H_14_	53-70-3	524	262	9.55 × 10^−10^	5.0 × 10^−4^	n.d.	2A	B2	511.4	n.d.
Benzo[g,h,i]perylene	C_22_H_12_	191-24-2	500	277	1.0 × 10^−10^	2.6 × 10^−5^	n.d.	3	D	517.1	n.d.
Indenol[1,2,3-cd]pyrene	C_22_H_12_	193-39-5	536	161-3	1.25 × 10^−3^	0.062	n.d.	2B	B2	349.2	n.d.

a *Data for vapor pressure, solubility, toxicology, and biodegradation has been adapted from Louvado et al. (2015) and the references therein*.

b *Mackay and Shiu (1977)*.

c *Toxic equivalent factor relatively to Benzo[a]pyrene (Chang et al., 2014)*.

d *International Agency for Research on Cancer Classification Monographs Volume 1–111 updated 18 February 2015 (1, carcinogenic to humans; 2A, probably carcinogenic to humans; 2B, possibly carcinogenic to humans; 3, not classifiable as carcinogenic to humans; n.c., not classified)*.

e *EPA carcinogenic classification: A, human carcinogenic; B1 and B2: probable human carcinogenic; C, possible human carcinogenic; D, not Classifiable as to human carcinogenicity; E, evidence of non-carcinogenicity for humans*.

f *Estimation using BioHCwin software v1.01 on EPI Suite software develop by Howard et al. (2005)*.

g *Comber et al. (2012); n.d., not determined*.

## Conflict of interest statement

The authors declare that the research was conducted in the absence of any commercial or financial relationships that could be construed as a potential conflict of interest.
