# Supplementary material for: Current State of Knowledge in Microbial Degradation of Polycyclic Aromatic Hydrocarbons (PAHs): A Review
Source: Front Microbiol. 2016 Aug 31;7:1369. doi: 10.3389/fmicb.2016.01369 (PMC5006600; doi:10.3389/fmicb.2016.01369)
Supplement: Supplementary file 1 [file Table_1.DOCX]

**Supplementary Table 1**: List of bacterial/archaeal (halophilic) strains involved in the degradation of PAHs.

| **Organism (Abbreviation)** | | | | **Substrate** | **References** |
| --- | --- | --- | --- | --- | --- |
| *Achromobacter xylosoxidans* (Axyl) | | | | Phn | Andreoni et al., 2004 |
| *Acidovorax delafieldii* P4-1 (AdelS45P1) | | | Phn | | Samanta et al., 1999 |
| *Acinetobacter* sp. (Acin) | | | Naph, Anth, Phn | | Ryu et al., 1989;  Lal and Khanna, 1996 |
| *Acinetobacter* sp. AGAT-W (AcinAGATW) | | | Acn, Acnl | | Ghosal et al., 2013 |
| *Actinopolyspora* sp. DPD15 (ActiDPD15) | | | Fl | | Al-Mueini et al., 2007 |
| *Aeromonas* sp. S45P1 (AeroS45P1) | Naph, Phn | | | | Kiyohara et al., 1976,  Kiyohara and Nagao, 1978 |
| *Agrobacterium* sp. (Agro) | | | Phn | | Aitken et al., 1998 |
| *Alcaligenes denitrificans* (Aden) | | | Fla | | Weissenfels et al., 1990 |
| *Alcaligenes faecalis* AFK2 (AfaeAFK2)  *Aquamicrobium defluvium* (Adef) | | | | Phn  Phn | Kiyohara et al., 1982  Andreoni et al., 2004 |
| *Arthrobacter* sp. F101 (Arth) | | | | Fl | Grifoll et al., 1992,  Casellas et al., 1997 |
| *Arthrobacter* sp. P1-1 (ArthP11) | | | | Phn | Seo et al., 2006 |
| *Arthrobacter sulphureus* RKJ4 (AsulRKJ4) | | | | Phn | Samanta et al., 1999 |
| *Bacillus cereus* P21 (BcerP21) | | | Phn, Pyr | | Aitken et al., 1998, Kazunga and Aitken, 2000 |
| *Bacillus* sp. DHT (Bcer) | | | Naph, Pyr | | Kumar et al., 2007 |
| *Bacillus thermoleovorans* Hamburg 2 (BtherHamburg2) | | | | Naph | Annweiler et al., 2000 |
| *Burkholderia cepacia* 2A-12 (Bcep) | | | Naph, Phn, Pyr | | Kim et al., 2003 |
| *Burkholderia* sp. BS3702 (BurkBS3702) | | | | Phn | Balashova et al., 1999 |
| *Burkholderia* sp. C3 (BurkC3) | | | | Phn | Seo et al., 2007 |
| *Burkholderia* sp. RP007 (BurkRP007) | | | | Naph, Phn | Laurie and Lloyd-Jones, 1999a; 1999b |
| *Clavibacter* *sp.* (Clav) | | | Naph | | Dore et al., 2003 |
| *Comamonas testosteroni* GZ38A (Ctes) | | Phn | | | Goyal and Zylstra, 1996 |
| *Comamonas testosteroni* GZ39 (Ctes) | | Phn | | | Goyal and Zylstra, 1996 |
| *Corynebacterium renale* ATCC 15075 (CrenATCC15075) | | Naph | | | Dua and Meera, 1981 |
| *Cycloclasticus* sp. A5 (CyclA5) | | | Naph, Anth, Phn, Fl | | Kasai et al. 2003; Geiselbrecht et al. 1998; Dyksterhouse et al., 1995 |
| *Cycloclasticus sp.* P1 (CyclP1) | | | Pyr | | Wang et al., 2008 |
| *Cycloclasticus* sp. PS-1 (CyclPS1) | | | Naph, Anth, Phn, Fl | | Kasai et al., 2003; Geiselbrecht et al., 1998;  Dyksterhouse et al., 1995 |
| *Cycloclasticus* sp. W (CyclW) | | | Naph, Anth, Phn, Fl | | Kasai et al., 2003; Geiselbrecht et al., 1998; Dyksterhouse et al., 1995 |
| *Enterobacter* sp. 214-6 (Ente2146) | | | Naph | | Toledo et al., 2006 |
| *Flavobacterium* sp. PJ711 (FlavPJ711) | | | Naph | | Widada et al,. 2002 |
| *Geobacillus* sp. G27 (GeobG27) | | | | Naph | Bubinas et al., 2008 |
| *Haloarcula hispanica* (Hhis) | | | Naph, Phn, Pyr | | Erdogmu¸s et al., 2013 |
| *Haloarcula* sp. (Hala) | | | Naph, Phn, Pyr | | Erdogmu¸s et al., 2013 |
| *Haloarcula vallismortis* EH4 (HvalEH4) | | | Acn, Phn, Anth | | Bertrand et al., 1990 |
| *Halobacterium piscisalsi* (Hpis) | | | Naph, Phn, Pyr | | Erdogmu¸s et al., 2013 |
| *Halobacterium salinarium* (Hsal) | | | Naph, Phn, Pyr | | Erdogmu¸s et al., 2013 |
| *Halobacterium* sp. (Halm) | | | Naph, Phn | | Al-Mailem et al., 2010 |
| *Halococcus* sp. (Hals) | | | Naph, Phn | | Al-Mailem et al., 2010 |
| *Haloferax* sp. (Halx) | | | Naph, Anth, Phn, Pyr | | Bonfá et al., 2011 |
| *Haloferax* sp. (Halx) | | | Naph, Phn, Pyr | | Erdogmu¸s et al., 2013 |
| *Haloferax* sp. (Halx) | | | Phn | | Tapilatu et al., 2010 |
| *Haloferax* sp. (Halx) | | | Naph, Phn | | Al-Mailem et al., 2010 |
| *Halorubrum ezzemoulense* (Hezz) | | | Naph, Phn, Pyr | | Erdogmu¸s et al., 2013 |
| *Halorubrum* sp. (Halo) | | | Naph, Phn, Pyr | | Erdogmu¸s et al., 2013 |
| *Janibacter* sp. YY-1 (JaniYY1) | | | Fl, Phn, Anth | | Yamazoe et al., 2004 |
| *Marinobacter falvimaris* (Mfal) | | | Naph, Anth, Phn | | Al-Mailem et al., 2013 |
| *Marinobacter nanhaiticus* (Mnan) | | | Naph, Anth, Phn | | Gao et al., 2013 |
| *Marinobacter sedimentalis* (Msed) | | | Naph, Anth, Phn | | Al-Mailem et al., 2013 |
| *Marinobacter* sp. NCE312 (MariNCE312) | | | Naph | | Hedlund et al., 2001 |
| *Methylobacterium* sp. (Meth) | | | Phn | | Andreoni et al., 2004 |
| *Micrococcus* sp. (Micr) | | | | Phn | Ghosh and Mishra, 1983 |
| *Moraxella* sp. Lav. 7 (MoraLav7) | | | | Naph | Tagger et al., 1990 |
| *Mycobacterium flavescens* (Mfla) | | | Phn, Fla, Pyr | | Dean-Ross and Cerniglia., 1996; Dean-Ross et al., 2002 |
| *Mycobacterium* sp. (Myco) | | | Pyr, Naph, Phn, Fla | | Heitkamp et al., 1988 |
| *Mycobacterium* sp. (Myco) | | | Pyr | | Grosser et al., 1991 |
| *Mycobacterium* sp. 6PY1 (Myco6PY1) | | | Pyr | | Krivobok et al., 2003 |
| *Mycobacterium* sp. Ap1 (Myco) | | | Pyr | | Vila et al., 2001 |
| *Mycobacterium* sp. BB1 (MycoBB1) | | | | Phn, Fl, Fla, Pyr | Boldrin et al., 1993 |
| *Mycobacterium* sp. BG1 (Myco) | | | | Phn | Guerin and Jones, 1988 |
| *Mycobacterium* sp. CH1 (Myco) | | | Phn, Fla, Pyr | | Churchill et al., 1999 |
| *Mycobacterium* sp. CH-2 (MycoCH-2) | | | Phn, Fla; Pyr | | Churchill et al., 2008 |
| *Mycobacterium* sp. JS14 (MycoJS14) | | | Fla | | Lee et al., 2007 |
| *Mycobacterium* sp. KMS (Myco) | | | Pyr | | Miller et al., 2004 |
| *Mycobacterium* sp. KR2 (Myco) | | | Pyr | | Rehmann et al., 1998 |
| *Mycobacterium* sp. KR20 (Myco) | | | Fla | | Rehmann et al., 2001 |
| *Mycobacterium* sp. LB501T (MycoLB501T) | | | | Anth | Van Herwijnen et al., 2003a |
| *Mycobacterium* sp. RJGII-135  (Myco) | | | Pyr, BaA, BaP | | Schneider et al., 1996 |
| *Mycobacterium vanbaalenii* PYR-1 (MvanPYR1) | | | | Naph, Anth, Phn, Fla, Pyr | Kelley et al., 1990; Moody et al., 2001; Kelley et al., 1993; Kim et al., 2005 |
| *Neptunomonas naphthovorans* NAG-2N-126 (NnapNAG2N126) | | | Naph | | Hedlund et al., 1999 |
| *Nocardia otitidiscaviarum*TSH1 (NotiTSH1) | | | | Naph, Anth, Phn | Zeinali et al., 2008a; 2008b |
| *Nocardioides* sp. KP7 (NocaKP7) | | | | Phn | Iwabuchi and Harayama 1997, Iwabuchi and Harayama, 1998 |
| *Novosphingobium naphthalenivorans* TUT562 (NnapTUT562) | | | Naph | | Suzuki and Hiraishi, 2007 |
| *Ochrobactrum* sp. PWTJD (OchrPWTJD) | | | Phn | | Ghosal et al., 2010 |
| *Paenibacillus naphthalenovorans* PR-N1 (PnapPRN1) | | | Naph | | Daane et al., 2001; 2002 |
| *Paenibacillus validus* DS-N1 (PvalDSN1) | | | Naph | | Daane et al., 2001; 2002 |
| *Paracoccus* sp. Ophe1 (ParaOphe1) | | | Anth, Phn, Fl | | Zhang et al., 2004 |
| *Pasteurella* sp. IFA (PastIFA) | | | Fla | | Sepic and Leskovsek., 1999 |
| *Polaromonas naphthalenivorans* CJ2 (PnapCJ2) | | | | Naph | Jeon et al., 2006 |
| *Pseudoalteromonas* sp. P-1P41 (PseuP1P41) | | | Naph, Phn, Fl | | Hedlund and Staley, 2006 |
| *Pseudomonas aeruginosa* (Paer) | | | Phn | | Romero et al., 1998 |
| *Pseudomonas aeruginosa* 2NR (Paer) | | | | Naph | Civilini et al., 1999 |
| *Pseudomonas aeruginosa* PaK1 (Paer) | | | | Naph | Takizawa et al., 1999 |
| *Pseudomonas aeruginosa* PF (Paer) | | | | Phn, Anth | Evans et al., 1965 |
| *Pseudomonas cepacia* F297 (Bcep) | | | | Fl | Grifoll et al., 1995 |
| *Pseudomonas fluorescens* (Pflu) | | | Chr, BaA | | Caldini et al., 1995 |
| *Pseudomonas fluorescens* LP6a (PfluLP6a) | | | | Naph | Foght and Westlake, 1996 |
| *Pseudomonas mendocina* CGMCC 1.766 (PmenCGMCC1766) | | | | Phn | Tian et al., 2003 |
| *Pseudomonas paucimobilis* (Ppau) | | | Phn | | Weissenfels et al., 1990 |
| *Pseudomonas putida* (Pput) | | | | Naph | Jeffrey et al., 1975 |
| *Pseudomonas putida* 9816 (Pput) | | | | Naph, Phn, Fl | Yang et al., 1994 |
| *Pseudomonas putida* BS3701 (Pput) | | | | Phn | Balashova et al., 1999 |
| *Pseudomonas putida* OUS82 (Pput) | | | | Naph, Phn, Anth | Kiyohara et al., 1994, Takizawa et al., 1999 |
| *Pseudomonas putida* PpG7 (PputPpG7) | | | | Naph | Dunn and Gunsalus, 1973 |
| *Pseudomonas saccharophila* P15 (Pseu) | | | Pyr | | Kazunga and Aitken, 2000 |
| *Pseudomonas* sp. (Pseu) | | | | Phn | Rogoff and Wender, 1957 |
| *Pseudomonas* sp. (Pseu) | | | | Naph | Davies and Evans, 1964 |
| *Pseudomonas* sp. (Pseu) | | | | Naph | Barnsley, 1975 |
| *Pseudomonas* sp. (Pseu) | | | | Acnl | Chapman, 1979 |
| *Pseudomonas* sp. C18 (Pseu) | | | Naph | | Denome et al., 1993 |
| *Pseudomonas* sp. DLC-P11 (Pseu) | | | | Phn | Samanta et al., 1999 |
| *Pseudomonas* sp. F274 (Pseu) | | | | Fl | Grifoll et al.,1994 |
| *Pseudomonas* sp. ND6 (PseuND6) | | | | Naph | Li et al., 2004 |
| *Pseudomonas* sp. PP2 (Pseu) | | | | Phn | Prabhu and Phale, 2003 |
| *Pseudomonas stutzeri* AN10 (PstuAN10) | | | | Naph | Bosch et al., 1999 |
| *Pseudomonas stutzeri* P16 (Pstu) | | | Pyr | | Kazunga and Aitken., 2000 |
| *Pusillimonas* sp. 21-11 (Pusi2111) | | | Naph, Phn | | Hilyard et al., 2008 |
| *Ralstonia* sp. U2 (RalsU2) | | | | Naph | Fuenmayor et al., 1998 |
| *Rhizobium* sp. CU-A1 (RhizCUA1) | | | | Acnl | Poonthrigpun et al., 2006 |
| *Rhodococcus opacus* R7 (RopaR7) | | | | Naph | Di Gennaro et al., 2001 |
| *Rhodococcus rhodochrous* VKM B-2469 (RrhoVKMB2469) | | | | Fl | Kolomytseva et al., 2009 |
| *Rhodococcus* sp. (Rhod) | | | | Anth, Fla | Dean-Ross et al., 2001; Dean-Ross et al., 2002 |
| *Rhodococcus* sp. B4 (Rhod) | | | | Naph | Grund et al., 1992 |
| *Rhodococcus* sp. UW1 (Rhod) | | | Pyr | | Walter et al., 1991 |
| *Rhodococcus* sp. NCIMB 12038 (RhodNCIMB12038) | | | | Naph | Allen et al., 1997 |
| *Shewanella* sp. 13-24 (Shew1324) | | | Naph | | Hilyard et al., 2008 |
| *Sinorhizobium* sp. C4 (SinoC4) | | | | Phn | Keum et al., 2006 |
| *Sphingobium chlorophenolicum* (Schl) | | | Naph, Phn | | Cavalca et al. 2007;  Chadhain et al., 2007 |
| *Sphingobium* sp. PNB (SphiPNB) | | | Naph, Phn, Anth | | Roy et al., 2012 |
| *Sphingobium yanoikuyae* B1 (SyanB1) | | | Naph, Phn | | Cavalca et al., 2007,  Chadhain et al., 2007 |
| *Sphingomonas paucimobilis* var. EPA505  (Spau) | | | Naph, Phn, Anth, Fla | | Story et al., 2001 |
| *Sphingomonas* sp. A4 (Sphi) | | | | Acn, Acnl | Pinyakong et al., 2004 |
| *Sphingomonas* sp. GY2B (SphiGY2B) | | | | Phn | Tao et al., 2007 |
| *Sphingomonas* sp. LB126 (SphiLB126) | | | | Fl, Phn, Anth, Fla | Wattiau et al., 2001;  Van Herwijnen et al., 2003b; 2003 |
| *Sphingomonas* sp. P2 (SphiP2) | | | | Phn | Pinyakong et al., 2000 |
| *Sphingomonas* sp. ZL5 (SphiZL5) | | | | Phn | Liu et al., 2004 |
| *Sphingomonas yanoikuyae* JAR02 (SyanJAR02) | | | BaP | | Rentz et al., 2008 |
| Sphingomonas yanoikuyae R1 (Syan) | | | Pyr | | Kazunga and Aitken, 2000 |
| *Staphylococcus auriculans* DBF63 (Stap) | | | | Fl | Monna et al., 1993 |
| *Staphylococcus* sp. PN/Y (StapPN/Y) | | | | Phn | Mallick et al., 2007 |
| *Stappia* sp. 23-41 (Stap2341) | | | Phn | | Hilyard et al., 2008 |
| *Vibrio cyclotrophicus* P-2P44 (VcycP2P44) | | | Naph, Phn | | Hedlund and Staley , 2001 |

Naph, naphthalene; Anth, anthracene; Phn, phenanthrene; Fl, Fluorene; Acn, acenaphthene; Acnl, acenaphthylene; Fla, fluoranthene; Pyr, pyrene; BaP, benzo[*a*]pyrene; BaA, benz[*a*]anthracene; DBA, dibenz[*a,h*]anthracene; Chr, chrysene.

**Supplementary Table 2**: List of cyanobacterial and algal (unicellular algae, multicellular algae and diatoms) strains employed in the bioremediation of PAHs.

| **Organism (Abbreviation)** | **Group** | **Substrate** | **References** |
| --- | --- | --- | --- |
| Agmenellum quadruplicatum*, strain* PR-6 (Agme) | Cyanobacteria | Naph, Phn | Cerniglia et al., 1979; Narro et al., 1992b; Cerniglia et al., 1980a |
| *Amphora sp., strain* AMP-1 (Amph) | Diatoms | Naph | Cerniglia et al., 1980a |
| *Anabaena fertilissima* (Anab) | Cyanobacteria | Anth, Pyr | Patel et al., 2016 |
| *Anabaena sp. strain* 1F (Anab) | Cyanobacteria | Naph | Cerniglia et al., 1980a |
| *Anabaena sp. strain* CA (Anab) | Cyanobacteria | Naph | Cerniglia et al., 1980a |
| *Ankistrodesmus braunii* (Anki) | Microalgae | BaP | Warshawsky et al., 1995 |
| *Aphanocapsa sp. strain* 6714 (Apha) | Cyanobacteria | Naph | Cerniglia et al., 1980a |
| *Chlamydomonas angulosa* (Chla) | Microalgae | Naph | Cerniglia et al., 1980a |
| *Chlamydomonas sp* (Chla) | Microalgae | Pyr | Lei et al., 2002 |
| *Chlorella autotrophica strain* 580 (Chlo) | Microalgae | Naph | Cerniglia et al., 1980a |
| Chlorella kessleri (Chlo) | Microalgae | BaP | Takáčová et al., 2014 |
| *Chlorella miniata* (Chlo) | Microalgae | Pyr | Lei et al., 2002 |
| Chlorella sorokiniana (Csor) | Microalgae | Phn | Muñoz et al., 2003 |
| *Chlorella sorokiniana* (Csor) | Microalgae | Phn | Borde et al., 2003 |
| *Chlorella sorokiniana strain* TX 71 105 (Csor) | Microalgae | Naph | Cerniglia et al., 1980a |
| *Chlorella vulgaris* (Cvul) | Microalgae | Pyr | Lei et al., 2002 |
| *Chlorella vulgaris* (Cvul) | Microalgae | Fla, Pyr | Lei et al., 2007 |
| *Chlorella vulgaris* (Cvul) | Microalgae | Naph, Anth | Sheekh et al., 2012 |
| Coccochloris elabens*, strain* 17A (Cocc) | Cyanobacteria | Naph | Cerniglia et al., 1979Cerniglia et al., 1980a |
| *Cylindrotheca sp., strain* N-1 (Cyli) | Diatomes | Naph | Cerniglia et al., 1980a |
| *Dunaliella tertiolecta strain* DUN (Dter) | Microalgae | Naph | Cerniglia et al., 1980a |
| *Elkatothrix viridis* (Evir) | Microalgae | Naph, Anth | Sheekh et al., 2012 |
| *Lyngbya lagerleritmi* (Lyng) | Microalgae | Naph, Anth | Sheekh et al., 2012 |
| *Microcoleus chthonoplastes strain* BA-1 (Mcht) | Cyanobacteria | Naph | Cerniglia et al., 1980a |
| *Nitzschia sp.* (Nitz) | Diatomes | Phn, Fla | Hong et al., 2008 |
| *Nostoc linckia* (Nost) |  | Naph | Sheekh et al., 2012 |
| *Nostoc sp. strain* MAC (Nost) | Cyanobacteria | Naph | Cerniglia et al., 1980a |
| *Oscillatoria rubescens* (Orub) | Cyanobacteria | Anth | Sheekh et al., 2012( |
| *Oscillatoria sp. strain* MEV (Osci) | Cyanobacteria | Naph | Cerniglia et al., 1980a |
| Oscillatoria *sp., strain* JCM (Osci) | Cyanobacteria | Naph | Cerniglia et al., 1979; Cerniglia et al., 1980a,b; Narro et al., 1992a |
| *Petalonia fascia* (Pfas) | Brown algae-multicellular | Naph | Cerniglia et al., 1980a |
| *Porphyridium cruentum* (Porp) | Red algae | Naph | Cerniglia et al., 1980a |
| *Prototheca zopfii* (Prot) | Microalgae | Naph, Phn, Pyr | Ueno et al., 2008 |
| *Scenedesmus acutus* (Scen) | Microalgae | BaP | Warshawsky et al., 1995 |
| Scenedesmus acutus (Scen) | Microalgae | BaP | de Llasera et al., 2016 |
| Scenedesmus obliquus ES-55 (Sobl) | Microalgae | Phn | Safonova et al., 2005 |
| *Scenedesmus platydiscus* (Scen) | Microalgae | Pyr | Lei et al., 2002 |
| *Scenedesmus platydiscus* (Scen) | Microalgae | Fla, Pyr | Lei et al., 2007 |
| *Scenedesmus quadricauda* (Squa) | Microalgae | Pyr | Lei et al., 2002 |
| *Scenedesmus quadricauda* (Squa) | Microalgae | Fla, Pyr | Lei et al., 2007 |
| *Selenastrum capricornutum* (Scap) | Microalgae | BaP | Warshawsky et al., 1988; Warshawsky et al., 1995 |
| *Selenastrum capricornutum* (Scap) | Microalgae | Pyr | Lei et al., 2002 |
| *Selenastrum capricornutum* (Scap) | Microalgae | Phn,  Fla, Pyr | Chan et al., 2006 |
| *Selenastrum capricornutum* (Scap) | Microalgae | Fla, Pyr | Lei et al., 2007 |
| Selenastrum capricornutum (Scap) | Microalgae | Fl, Phn, Fla,  Pyr, BaP | Ke et al., 2010 |
| *Selenastrum capricornutum* (Scap) | Microalgae | BaA and BaP | Luo et al., 2014 |
| Selenastrum capricornutum (Scap) | Microalgae | BaP | de Llasera et al., 2016 |
| *Skeletonema costatum* (Skel) | Microalgae | Phn, Fla | Hong et al., 2008 |
| *Synechosystis sp* (Syne) | Cyanobacteria | Pyr | Lei et al., 2002 |
| *Ulva fasciata* (Ulva) | Green algae- multicellular | Naph | Cerniglia et al., 1980a |
| *Volvox aureus* (Volv) | Microalgae | Naph | Sheekh et al., 2012 |

Naph, naphthalene; Anth, anthracene; Phn, phenanthrene; Fl, Fluorene; Fla, fluoranthene; Pyr, pyrene; BaP, benzo[*a*]pyrene; BaA, benz[*a*]anthracene.

**REFERENCES:**

Aitken, M.D., Stringfellow, W.T., Nagel, R.D., Kazunga, C., and Chen, S.H. (1998). Characteristics of phenanthrene-degrading bacteria isolated from soils contaminated with polycyclic aromatic hydrocarbons. *Can J Microbiol* 44(8)**,** 743-752.

Al-Mailem, D.M., Eliyas, M., and Radwan, S.S. (2013). Oil-bioremediation potential of two hydrocarbonoclastic, diazotrophic Marinobacter strains from hypersaline areas along the Arabian Gulf coasts. . *Extremophiles* 17**,** 463-470.

Al-Mailem, D.M., Sorkhoh, N.A., Al-Awadhi, H., Eliyas, M., and Radwan, S.S. (2010). Biodegradation of crude oil and pure hydrocarbons by extreme halophilic archaea from hypersaline coasts of the Arabian Gulf. *Extremophiles* 14**,** 321-328.

Al-Mueini, R., Al-Dalali, M., Al-Amri, I.S., and Patzelt, H. (2007). Hydrocarbon degradation at high salinity by a novel extremely halophilic actinomycete. . *Environ. Chem.* 4**,** 5-7.

Andreoni, V., Cavalca, L., Rao, M.A., Nocerino, G., Bernasconi, S., Dell'Amico, E., et al. (2004). Bacterial communities and enzyme activities of PAHs polluted soils. *Chemosphere* 57(5)**,** 401-412. doi: 10.1016/j.chemosphere.2004.06.013.

Annweiler, E., Richnow, H.H., Antranikian, G., Hebenbrock, S., Garms, C., Franke, S., et al. (2000). Naphthalene degradation and incorporation of naphthalene-derived carbon into biomass by the thermophile *Bacillus thermoleovorans*. *Appl Environ Microbiol* 66(2)**,** 518-523.

Balashova, N.V., Kosheleva, I.A., Golovchenko, N.P., and Boronin, A.M. (1999). Phenanthrene metabolism by *Pseudomonas* and *Burkholderia* strains. *Proc Biochem.* 35**,** 291-296.

Barnsley, E.A. (1975). The induction of the enzymes of naphthalene metabolism in pseudomonads by salicylate and 2-aminobenzoate. *J Gen Microbiol* 88(1)**,** 193-196. doi: 10.1099/00221287-88-1-193.

Bertrand, J.C., Almallah, M., Acquaviva, M., and Mille, G. (1990). Biodegradation of hydrocarbons by an extremely halophilic archaebacterium. . *Lett.Appl. Microbiol.* 11**,** 260-263.

Boldrin, B., Tiehm, A., and Fritzsche, C. (1993). Degradation of phenanthrene, fluorene, fluoranthene, and pyrene by a *Mycobacterium* sp. *Appl Environ Microbiol* 59(6)**,** 1927-1930.

Bonfa, M.R., Grossman, M.J., Mellado, E., and Durrant, L.R. (2011). Biodegradation of aromatic hydrocarbons by Haloarchaea and their use for the reduction of the chemical oxygen demand of hypersaline petroleum produced water. *Chemosphere* 84(11)**,** 1671-1676. doi: 10.1016/j.chemosphere.2011.05.005.

Borde, X., Guieysse, B., Delgado, O., Munoz, R., Hatti-Kaul, R., Nugier-Chauvin, C., et al. (2003). Synergistic relationships in algal-bacterial microcosms for the treatment of aromatic pollutants. *Bioresour Technol* 86(3)**,** 293-300.

Bosch, R., Garcia-Valdes, E., and Moore, E.R. (1999). Genetic characterization and evolutionary implications of a chromosomally encoded naphthalene-degradation upper pathway from *Pseudomonas stutzeri* AN10. *Gene* 236(1)**,** 149-157.

Boyd, C., Larkin, M.J., Reid, K.A., Sharma, N.D., and Wilson, K. (1997). Metabolism of Naphthalene, 1-Naphthol, Indene, and Indole by *Rhodococcus* sp. Strain NCIMB 12038. *Appl Environ Microbiol* 63(1)**,** 151-155.

Bubinas, A., Giedraityte, G., Kalediene, L., Nivinakiene, O., and Butkiene, R. (2008). Degradation of naphthalene by thermophilic bacteria via a pathway, through protocatechuic acid. *Cent Eur J Biol.* 3**,** 61-68.

Caldini, G., Cenci, G., Manenti, R., and Morozzi, G. (1995). The ability of an environmental isolate of *Pseudomonas fluorescens* to utilize chrysene and other four-ring polycyclic aromatic hydrocarbons. . *Appl. Microbiol. Biotechnol.* 44**,** 225-229.

Casellas, M., Grifoll, M., Bayona, J.M., and Solanas, A.M. (1997). New metabolites in the degradation of fluorene by *Arthrobacter* sp. strain F101. *Appl Environ Microbiol* 63(3)**,** 819-826.

Cavalca, L., Guerrieri, N., Colombo, M., Pagani, S., and Andreoni, V. (2007). Enzymatic and genetic profiles in environmental strains grown on polycyclic aromatic hydrocarbons. *Antonie Van Leeuwenhoek* 91(4)**,** 315-325. doi: 10.1007/s10482-006-9119-1.

Cerniglia, C.E., Baalen, C.V., and Gibson, D.T. (1980b). Metabolism of naphthalene by cyanobacterium *Oscillatoria* sp. Strain JCM. *J. Gen. Microbiol.* 116**,** 485-494.

Cerniglia, C.E., Gibson, D.T., and Van Baalen, C. (1979). Algal oxidation of aromatic hydrocarbons: formation of 1-naphthol from naphthalene by *Agmenellum quadruplicatum*, strain PR-6. *Biochem Biophys Res Commun* 88(1)**,** 50-58.

Cerniglia, C.E., Gibson, D.T., and Van Baalen, C. (1980a). Oxidation of naphthalene by cyanobacteria and microalgae. *J. Gen. Microbiol.* 116**,** 495-500.

Chadhain, S.M., Moritz, E.M., Kim, E., and Zylstra, G.J. (2007). Identification, cloning, and characterization of a multicomponent biphenyl dioxygenase from *Sphingobium yanoikuyae* B1. *J Ind Microbiol Biotechnol* 34(9)**,** 605-613. doi: 10.1007/s10295-007-0235-3.

Chan, S.M., Luan, T., Wong, M.H., and Tam, N.F. (2006). Removal and biodegradation of polycyclic aromatic hydrocarbons by *Selenastrum capricornutum*. *Environ Toxicol Chem.* 7**,** 1772-1779.

Chapman, P.J. (1979). Degradation mechanisms. In: Bourquin AW, Pritchard PH, eds. Proceedings of the workshop: microbial degradation of pollutants in marine environments. . *Gulf Breeze: U.S. Environmental Protection Agency.***,** 28-66.

Churchill, P.F., Morgan, A.C., and Kitchens, E. (2008). Characterization of a pyrene-degrading *Mycobacterium* sp. strain CH-2. *J Environ Sci Health B* 43(8)**,** 698-706. doi: 10.1080/03601230802388801.

Churchill, S.A., Harper, J.P., and Churchill, P.F. (1999). Isolation and characterization of a *Mycobacterium* species capable of degrading three- and four-ring aromatic and aliphatic hydrocarbons. *Appl Environ Microbiol* 65(2)**,** 549-552.

Civilini, M., de Bertoldi, M., and Tell, G. (1999). Molecular characterization of *Pseudomonas aeruginosa* 2NR degrading naphthalene. *Lett Appl Microbiol* 29(3)**,** 181-186.

Daane, L.L., Harjono, I., Barns, S.M., Launen, L.A., Palleron, N.J., and Haggblom, M.M. (2002). PAH-degradation by Paenibacillus spp. and description of *Paenibacillus naphthalenovorans* sp. nov., a naphthalene-degrading bacterium from the rhizosphere of salt marsh plants. *Int J Syst Evol Microbiol* 52(Pt 1)**,** 131-139. doi: 10.1099/00207713-52-1-131.

Daane, L.L., Harjono, I., Zylstra, G.J., and Haggblom, M.M. (2001). Isolation and characterization of polycyclic aromatic hydrocarbon-degrading bacteria associated with the rhizosphere of salt marsh plants. *Appl Environ Microbiol* 67(6)**,** 2683-2691. doi: 10.1128/AEM.67.6.2683-2691.2001.

Davies, J.I., and Evans, W.C. (1964). Oxidative metabolism of naphthalene by soil pseudomonads. The ring-fission mechanism. *Biochem J* 91(2)**,** 251-261.

Dean-Ross, D., and Cerniglia, C.E. (1996). Degradation of pyrene by *Mycobacterium flavescens*. *Appl Microbiol Biotechnol* 46(3)**,** 307-312.

Dean-Ross, D., Moody, J., and Cerniglia, C.E. (2002). Utilization of mixtures of polycyclic aromatic hydrocarbons by bacteria isolated from contaminated sediment. *FEMS Microbiol Ecol* 41(1)**,** 1-7. doi: 10.1111/j.1574-6941.2002.tb00960.x.

Dean-Ross, D., Moody, J.D., Freeman, J.P., Doerge, D.R., and Cerniglia, C.E. (2001). Metabolism of anthracene by a *Rhodococcus* species. *FEMS Microbiol Lett* 204(1)**,** 205-211.

Denome, S.A., Stanley, D.C., Olson, E.S., and Young, K.D. (1993). Metabolism of dibenzothiophene and naphthalene in *Pseudomonas* strains: complete DNA sequence of an upper naphthalene catabolic pathway. *J Bacteriol* 175(21)**,** 6890-6901.

Di Gennaro, P., Rescalli, E., Galli, E., Sello, G., and Bestetti, G. (2001). Characterization of *Rhodococcus opacus* R7, a strain able to degrade naphthalene and o-xylene isolated from a polycyclic aromatic hydrocarbon-contaminated soil. *Res Microbiol* 152(7)**,** 641-651.

Dore, S.Y., Clancy, Q.E., Rylee, S.M., and Kulpa, C.F., Jr. (2003). Naphthalene-utilizing and mercury-resistant bacteria isolated from an acidic environment. *Appl Microbiol Biotechnol* 63(2)**,** 194-199. doi: 10.1007/s00253-003-1378-4.

Dua, R.D., and Meera, S. (1981). Purification and characterisation of naphthalene oxygenase from *Corynebacterium renale*. *Eur J Biochem* 120(3)**,** 461-465.

Dunn, N.W., and Gunsalus, I.C. (1973). Transmissible plasmid coding early enzymes of naphthalene oxidation in *Pseudomonas putida*. *J Bacteriol* 114(3)**,** 974-979.

Dyksterhouse, S.E., Gray, J.P., Herwig, R.P., Lara, J.C., and Staley, J.T. (1995). *Cycloclasticus pugetii* gen. nov., sp. nov., an aromatic hydrocarbon-degrading bacterium from marine sediments. *Int J Syst Bacteriol* 45(1)**,** 116-123. doi: 10.1099/00207713-45-1-116.

El-Sheekh, M.M., Ghareib, M.M., and Abou-EL-Souod, G.W. (2012). Biodegradation of Phenolic and Polycyclic Aromatic Compounds by Some Algae and Cyanobacteria. . *J Bioremed Biodegrad* 3(1).

Erdogmuş, S.F., Mutlu, B., Korcan, S.E., Guven, K., and Konuk, M. (2013). Aromatic hydrocarbon degradation by halophilic archaea isolated from Çamalti Saltern, Turkey. *Water Air Soil Pollut.* 224**,** 1449-1449.

Evans, W.C., Fernley, H.N., and Griffiths, E. (1965). Oxidative Metabolism of Phenanthrene and Anthracene by Soil Pseudomonads. The Ring-Fission Mechanism. *Biochem J* 95**,** 819-831.

Foght, J.M., and Westlake, D.W. (1996). Transposon and spontaneous deletion mutants of plasmid-borne genes encoding polycyclic aromatic hydrocarbon degradation by a strain of *Pseudomonas fluorescens*. *Biodegradation* 7(4)**,** 353-366.

Fuenmayor, S.L., Wild, M., Boyes, A.L., and Williams, P.A. (1998). A gene cluster encoding steps in conversion of naphthalene to gentisate in *Pseudomonas* sp. strain U2. *J Bacteriol* 180(9)**,** 2522-2530.

Gao, W., Cui, Z., Li, Q., Xu, G., Jia, X., and Zheng, L. (2013). *Marinobacter nanhaiticus* sp. nov., polycyclic aromatic hydrocarbon-degrading bacterium isolated from the sediment of the South China Sea. *Antonie Van Leeuwenhoek* 103(3)**,** 485-491. doi: 10.1007/s10482-012-9830-z.

Garcia de Llasera, M.P., Olmos-Espejel Jde, J., Diaz-Flores, G., and Montano-Montiel, A. (2016). Biodegradation of benzo(a)pyrene by two freshwater microalgae *Selenastrum capricornutum* and *Scenedesmus acutus*: a comparative study useful for bioremediation. *Environ Sci Pollut Res Int* 23(4)**,** 3365-3375. doi: 10.1007/s11356-015-5576-2.

Geiselbrecht, A.D., Hedlund, B.P., Tichi, M.A., and Staley, J.T. (1998). Isolation of marine polycyclic aromatic hydrocarbon (PAH)-degrading *Cycloclasticus* strains from the Gulf of Mexico and comparison of their PAH degradation ability with that of puget sound Cycloclasticus strains. *Appl Environ Microbiol* 64(12)**,** 4703-4710.

Ghosal, D., Chakraborty, J., Khara, P., and Dutta, T.K. (2010). Degradation of phenanthrene via meta-cleavage of 2-hydroxy-1-naphthoic acid by *Ochrobactrum* sp. strain PWTJD. *FEMS Microbiol Lett* 313(2)**,** 103-110. doi: 10.1111/j.1574-6968.2010.02129.x.

Ghosal, D., Dutta, A., Chakraborty, J., Basu, S., and Dutta, T.K. (2013). Characterization of the metabolic pathway involved in assimilation of acenaphthene in *Acinetobacter* sp. strain AGAT-W. *Res Microbiol* 164(2)**,** 155-163. doi: 10.1016/j.resmic.2012.11.003.

Ghosh, D.K., and Mishra, A.K. (1983). Oxidation of phenanthrene by a strain of *Micrococcus*: Evidence of protocatechuate pathway. *Curr Microbiol.* 9**,** 219-224.

Goyal, A.K., and Zylstra, G.J. (1996). Molecular cloning of novel genes for polycyclic aromatic hydrocarbon degradation from *Comamonas testosteroni* GZ39. *Appl Environ Microbiol* 62(1)**,** 230-236.

Grifoll, M., Casellas, M., Bayona, J.M., and Solanas, A.M. (1992). Isolation and characterization of a fluorene-degrading bacterium: identification of ring oxidation and ring fission products. *Appl Environ Microbiol* 58(9)**,** 2910-2917.

Grifoll, M., Selifonov, S.A., and Chapman, P.J. (1994). Evidence for a novel pathway in the degradation of fluorene by *Pseudomonas* sp. strain F274. *Appl Environ Microbiol* 60(7)**,** 2438-2449.

Grifoll, M., Selifonov, S.A., Gatlin, C.V., and Chapman, P.J. (1995). Actions of a versatile fluorene-degrading bacterial isolate on polycyclic aromatic compounds. *Appl Environ Microbiol* 61(10)**,** 3711-3723.

Grosser, R.J., Warshawsky, D., and Vestal, J.R. (1991). Indigenous and enhanced mineralization of pyrene, benzo[a]pyrene, and carbazole in soils. *Appl Environ Microbiol* 57(12)**,** 3462-3469.

Grund, E., Denecke, B., and Eichenlaub, R. (1992). Naphthalene degradation via salicylate and gentisate by *Rhodococcus* sp. strain B4. *Appl Environ Microbiol* 58(6)**,** 1874-1877.

Guerin, W.F., and Jones, G.E. (1988). Mineralization of phenanthrene by a *Mycobacterium* sp. *Appl Environ Microbiol* 54(4)**,** 937-944.

Hedlund, B.P., Geiselbrecht, A.D., Bair, T.J., and Staley, J.T. (1999). Polycyclic aromatic hydrocarbon degradation by a new marine bacterium, *Neptunomonas naphthovorans* gen. nov., sp. nov. *Appl Environ Microbiol* 65(1)**,** 251-259.

Hedlund, B.P., Geiselbrecht, A.D., and Staley, J.T. (2001). *Marinobacter* strain NCE312 has a Pseudomonas-like naphthalene dioxygenase. *FEMS Microbiol Lett* 201(1)**,** 47-51.

Hedlund, B.P., and Staley, J.T. (2006). Isolation and characterization of *Pseudoalteromonas* strains with divergent polycyclic aromatic hydrocarbon catabolic properties. *Environ Microbiol* 8(1)**,** 178-182. doi: 10.1111/j.1462-2920.2005.00871.x.

Heitkamp, M.A., and Cerniglia, C.E. (1988). Mineralization of polycyclic aromatic hydrocarbons by a bacterium isolated from sediment below an oil field. *Appl Environ Microbiol* 54(6)**,** 1612-1614.

Herwijnen, R., Sande, B.F., Wielen, F.W., Springael, D., Govers, H.A., and Parsons, J.R. (2003). Influence of phenanthrene and fluoranthene on the degradation of fluorene and glucose by *Sphingomonas* sp. strain LB126 in chemostat cultures. *FEMS Microbiol Ecol* 46(1)**,** 105-111. doi: 10.1016/S0168-6496(03)00202-2.

Hilyard, E.J., Jones-Meehan, J.M., Spargo, B.J., and Hill, R.T. (2008). Enrichment, isolation, and phylogenetic identification of polycyclic aromatic hydrocarbon-degrading bacteria from Elizabeth River sediments. *Appl Environ Microbiol* 74(4)**,** 1176-1182. doi: 10.1128/AEM.01518-07.

Hong, Y.W., Yuan, D.X., Lin, Q.M., and Yang, T.L. (2008). Accumulation and biodegradation of phenanthrene and fluoranthene by the algae enriched from a mangrove aquatic ecosystem. *Mar Pollut Bull* 56(8)**,** 1400-1405. doi: 10.1016/j.marpolbul.2008.05.003.

Iwabuchi, T., and Harayama, S. (1997). Biochemical and genetic characterization of 2-carboxybenzaldehyde dehydrogenase, an enzyme involved in phenanthrene degradation by *Nocardioides* sp. Strain KP7. . *J Bacteriol.* 179**,** 6488–6494.

Iwabuchi, T., and Harayama, S. (1998). Biochemical and genetic characterization of trans-2'-carboxybenzalpyruvate hydratase-aldolase from a phenanthrene-degrading *Nocardioides* strain. . *J Bacteriol.* 180**,** 945-949.

Jeffrey, A.M., Yeh, H.J., Jerina, D.M., Patel, T.R., Davey, J.F., and Gibson, D.T. (1975). Initial reactions in the oxidation of naphthalene by *Pseudomonas putida*. *Biochemistry* 14(3)**,** 575-584.

Jeon, C.O., Park, M., Ro, H.S., Park, W., and Madsen, E.L. (2006). The naphthalene catabolic (nag) genes of *Polaromonas naphthalenivorans* CJ2: evolutionary implications for two gene clusters and novel regulatory control. *Appl Environ Microbiol* 72(2)**,** 1086-1095. doi: 10.1128/AEM.72.2.1086-1095.2006.

Kasai, Y., Shindo, K., Harayama, S., and Misawa, N. (2003). Molecular characterization and substrate preference of a polycyclic aromatic hydrocarbon dioxygenase from *Cycloclasticus* sp. strain A5. *Appl Environ Microbiol* 69(11)**,** 6688-6697.

Kazunga, C., and Aitken, M.D. (2000). Products from the incomplete metabolism of pyrene by polycyclic aromatic hydrocarbon-degrading bacteria. *Appl Environ Microbiol* 66(5)**,** 1917-1922.

Ke, L., Luo, L., Wang, P., Luan, T., and Tam, N.F. (2010). Effects of metals on biosorption and biodegradation of mixed polycyclic aromatic hydrocarbons by a freshwater green alga *Selenastrum capricornutum*. *Bioresour Technol* 101(18)**,** 6961-6972. doi: 10.1016/j.biortech.2010.04.011.

Kelley, I., Freeman, J.P., and Cerniglia, C.E. (1990). Identification of metabolites from degradation of naphthalene by a *Mycobacterium* sp. *Biodegradation* 1(4)**,** 283-290.

Keum, Y.S., Seo, J.S., Hu, Y., and Li, Q.X. (2006). Degradation pathways of phenanthrene by *Sinorhizobium* sp. C4. *Appl Microbiol Biotechnol* 71(6)**,** 935-941. doi: 10.1007/s00253-005-0219-z.

Kim, T.J., Lee, E.Y., Kim, Y.J., Cho, K.S., and Ryu, H.W. (2003). Degradation of polyaromatic hydrocarbons by *Burkholderia cepacia* 2A-12. . *World J. Microbiol. Biotechnol.* 19**,** 411-417.

Kim, Y.H., Freeman, J.P., Moody, J.D., Engesser, K.H., and Cerniglia, C.E. (2005). Effects of pH on the degradation of phenanthrene and pyrene by *Mycobacterium vanbaalenii* PYR-1. *Appl Microbiol Biotechnol* 67(2)**,** 275-285. doi: 10.1007/s00253-004-1796-y.

Kiyohara, H., and Nagao, K. (1978). The catabolism of phenanthrene and naphthalene by bacteria. *J Gen Microbiol.* 105**,** 69–75

Kiyohara, H., Nagao, K., Kouno, K., and Yano, K. (1982). Phenanthrene degrading phenotype of *Alcaligenes faecalis* AFK2. . *Appl Environ Microbiol.* 43**,** 458–461.

Kiyohara, H., Nagao, K., and Nomi, R. (1976). Degradation of phenanthrene through o-phthalate by an Aeromonas sp. . *Agric Biol Chem.* 40**,** 1075-1082.

Kiyohara, H., Torigoe, S., Kaida, N., Asaki, T., Iida, T., Hayashi, H., et al. (1994). Cloning and characterization of a chromosomal gene cluster, pah, that encodes the upper pathway for phenanthrene and naphthalene utilization by *Pseudomonas putida* OUS82. *J Bacteriol* 176(8)**,** 2439-2443.

Kolomytseva, M.P., Randazzo, D., Baskunov, B.P., Scozzafava, A., Briganti, F., and Golovleva, L.A. (2009). Role of surfactants in optimizing fluorene assimilation and intermediate formation by *Rhodococcus rhodochrous* VKM B-2469. *Bioresour Technol* 100(2)**,** 839-844. doi: 10.1016/j.biortech.2008.06.059.

Krivobok, S., Kuony, S., Meyer, C., Louwagie, M., Willison, J.C., and Jouanneau, Y. (2003). Identification of pyrene-induced proteins in *Mycobacterium* sp. strain 6PY1: evidence for two ring-hydroxylating dioxygenases. *J Bacteriol* 185(13)**,** 3828-3841.

Kumar, M., Vladimir, L., de Sistro Materano, A., and Ilzins, O.A. (2007). A halotolerant and thermotolerant *Bacillus* sp. degrades hydrocarbons and produces tension active emulsifying agent. . *World J. Microbiol. Biotechnol.* 23**,** 211-220.

Lal, B., and Khanna, S. (1996). Degradation of crude oil by *Acinetobacter calcoaceticus* and *Alcaligenes odorans*. *J Appl Bacteriol* 81(4)**,** 355-362.

Laurie, A.D., and Lloyd-Jones, G. (1999a). The phn genes of *Burkholderia* sp. strain RP007 constitute a divergent gene cluster for polycyclic aromatic hydrocarbon catabolism. *J Bacteriol* 181(2)**,** 531-540.

Laurie, A.D., and Lloyd-Jones, G. (1999b). Conserved and hybrid meta-cleavage operons from PAH-degrading *Burkholderia* RP007. *Biochem Biophys Res Commun* 262(1)**,** 308-314. doi: 10.1006/bbrc.1999.1153.

Lee, S.E., Seo, J.S., Keum, Y.S., Lee, K.J., and Li, Q.X. (2007). Fluoranthene metabolism and associated proteins in Mycobacterium sp. JS14. *Proteomics* 7(12)**,** 2059-2069. doi: 10.1002/pmic.200600489.

Lei, A.P., Hu, Z.L., Wong, Y.S., and Tam, N.F. (2007). Removal of fluoranthene and pyrene by different microalgal species. *Bioresour Technol* 98(2)**,** 273-280. doi: 10.1016/j.biortech.2006.01.012.

Lei, A.P., Wong, Y.S., and Tam, N.F. (2002). Removal of pyrene by different microalgal species. *Water Sci Technol* 46(11-12)**,** 195-201.

Li, W., Shi, J., Wang, X., Han, Y., Tong, W., Ma, L., et al. (2004). Complete nucleotide sequence and organization of the naphthalene catabolic plasmid pND6-1 from *Pseudomonas* sp. strain ND6. *Gene* 336(2)**,** 231-240. doi: 10.1016/j.gene.2004.03.027.

Liu, Y., Zhang, J., and Zhang, Z. (2004). Isolation and characterization of polycyclic aromatic hydrocarbons-degrading *Sphingomonas* sp. strain ZL5. *Biodegradation* 15(3)**,** 205-212.

Luo, L.W., P.; Lin, L.; Luan, T.; Ke, L.; and Tam, NFY. (2014). Removal and transformation of high molecular weight polycyclic aromatic hydrocarbons in water by live and dead microalgae. *Process Biochemistry.* 49(10)**,** 1723–1732.

Mallick, S., Chatterjee, S., and Dutta, T.K. (2007). A novel degradation pathway in the assimilation of phenanthrene by *Staphylococcus* sp. strain PN/Y via meta-cleavage of 2-hydroxy-1-naphthoic acid: formation of trans-2,3-dioxo-5-(2'-hydroxyphenyl)-pent-4-enoic acid. *Microbiology* 153(Pt 7)**,** 2104-2115. doi: 10.1099/mic.0.2006/004218-0.

Miller, C.D., Hall, K., Liang, Y.N., Nieman, K., Sorensen, D., Issa, B., et al. (2004). Isolation and characterization of polycyclic aromatic hydrocarbon-degrading *Mycobacterium* isolates from soil. *Microb Ecol* 48(2)**,** 230-238. doi: 10.1007/s00248-003-1044-5.

Monna, L., Omori, T., and Kodama, T. (1993). Microbial degradation of dibenzofuran, fluorene, and dibenzo-p-dioxin by *Staphylococcus auriculans* DBF63. *Appl Environ Microbiol* 59(1)**,** 285-289.

Moody, J.D., Freeman, J.P., Doerge, D.R., and Cerniglia, C.E. (2001). Degradation of phenanthrene and anthracene by cell suspensions of *Mycobacterium* sp. strain PYR-1. *Appl Environ Microbiol* 67(4)**,** 1476-1483. doi: 10.1128/AEM.67.4.1476-1483.2001.

Munoz, R., Guieysse, B., and Mattiasson, B. (2003). Phenanthrene biodegradation by an algal-bacterial consortium in two-phase partitioning bioreactors. *Appl Microbiol Biotechnol* 61(3)**,** 261-267. doi: 10.1007/s00253-003-1231-9.

Narro, M.L., Cerniglia, C.E., Van Baalen, C., and Gibson, D.T. (1992a). Evidence for an NIH shift in oxidation of naphthalene by the marine cyanobacterium *Oscillatoria* sp. strain JCM. *Appl Environ Microbiol* 58(4)**,** 1360-1363.

Narro, M.L., Cerniglia, C.E., Van Baalen, C., and Gibson, D.T. (1992b). Metabolism of phenanthrene by the marine cyanobacterium *Agmenellum quadruplicatum* PR-6. *Appl Environ Microbiol* 58(4)**,** 1351-1359.

Patel, J.G., Nirmal Kumar, J.I., Kumar, R.N., and Khan, S.R. (2016). Biodegradation Capability and Enzymatic Variation of Potentially Hazardous Polycyclic Aromatic Hydrocarbons—Anthracene and Pyrene by *Anabaena fertilissima*. *Polycyclic Aromatic Compounds.* 36(1)**,** 72-87.

Pinyakong, O., Habe, H., Kouzuma, A., Nojiri, H., Yamane, H., and Omori, T. (2004). Isolation and characterization of genes encoding polycyclic aromatic hydrocarbon dioxygenase from acenaphthene and acenaphthylene degrading *Sphingomonas* sp. strain A4. *FEMS Microbiol Lett* 238(2)**,** 297-305. doi: 10.1016/j.femsle.2004.07.048.

Pinyakong, O., Habe, H., Supaka, N., Pinpanichkarn, P., Juntongjin, K., Yoshida, T., et al. (2000). Identification of novel metabolites in the degradation of phenanthrene by *Sphingomonas* sp. strain P2. *FEMS Microbiol Lett* 191(1)**,** 115-121.

Poonthrigpun, S., Pattaragulwanit, K., Paengthai, S., Kriangkripipat, T., Juntongjin, K., Thaniyavarn, S., et al. (2006). Novel intermediates of acenaphthylene degradation by *Rhizobium* sp. strain CU-A1: evidence for naphthalene-1,8-dicarboxylic acid metabolism. *Appl Environ Microbiol* 72(9)**,** 6034-6039. doi: 10.1128/AEM.00897-06.

Prabhu, Y., and Phale, P.S. (2003). Biodegradation of phenanthrene by *Pseudomonas* sp. strain PP2: novel metabolic pathway, role of biosurfactant and cell surface hydrophobicity in hydrocarbon assimilation. *Appl Microbiol Biotechnol* 61(4)**,** 342-351. doi: 10.1007/s00253-002-1218-y.

Rehmann, K., Hertkorn, N., and Kettrup, A.A. (2001). Fluoranthene metabolism in *Mycobacterium* sp. strain KR20: identity of pathway intermediates during degradation and growth. *Microbiology* 147(Pt 10)**,** 2783-2794. doi: 10.1099/00221287-147-10-2783.

Rehmann, K., Noll, H.P., Steinberg, C.E., and Kettrup, A.A. (1998). Pyrene degradation by *Mycobacterium* sp. strain KR2. *Chemosphere* 36(14)**,** 2977-2992.

Rentz, J.A., Alvarez, P.J., and Schnoor, J.L. (2008). Benzo[a]pyrene degradation by *Sphingomonas yanoikuyae* JAR02. *Environ Pollut* 151(3)**,** 669-677. doi: 10.1016/j.envpol.2007.02.018.

Rogoff, M.H., and Wender, I. (1957). The microbiology of coal. I. Bacterial oxidation of phenanthrene. *J Bacteriol* 73(2)**,** 264-268.

Romero, M.C., Cazau, M.C., Giorgieri, S., and Arambarri, A.M. (1998). Phenanthrene degradation by microorganisms isolated from a contaminated stream. *Environ. Pollut.* 101**,** 355-359.

Roy, M., Khara, P., and Dutta, T.K. (2012). meta-Cleavage of hydroxynaphthoic acids in the degradation of phenanthrene by *Sphingobium* sp. strain PNB. *Microbiology* 158(Pt 3)**,** 685-695. doi: 10.1099/mic.0.053363-0.

Ryu, B.H., Oh, Y.K., and Bin, J.H. (1989). Biodegradation of naphthalene by *Acinetobacter calcoaceticus* R-88. . *J Kor Agric Chem Soc,* 32**,** 315-320.

Safonova, E.K., K.; Kuschk, P.; Möder, M.; and Reisser, W. (2005). Biodegradation of Phenanthrene by the Green Alga *Scenedesmus obliquus* ES-55. *Eng. Life Sci.* **,** 234-239.

Samanta, S.K., Chakraborti, A.K., and Jain, R.K. (1999). Degradation of phenanthrene by different bacteria: evidence for novel transformation sequences involving the formation of 1-naphthol. *Appl Microbiol Biotechnol* 53(1)**,** 98-107.

Schneider, J., Grosser, R., Jayasimhulu, K., Xue, W., and Warshawsky, D. (1996). Degradation of pyrene, benz[a]anthracene, and benzo[a]pyrene by *Mycobacterium* sp. strain RJGII-135, isolated from a former coal gasification site. *Appl Environ Microbiol* 62(1)**,** 13-19.

Seo, J.S., Keum, Y.S., Hu, Y., Lee, S.E., and Li, Q.X. (2006). Phenanthrene degradation in *Arthrobacter* sp. P1-1: initial 1,2-, 3,4- and 9,10-dioxygenation, and meta- and ortho-cleavages of naphthalene-1,2-diol after its formation from naphthalene-1,2-dicarboxylic acid and hydroxyl naphthoic acids. *Chemosphere* 65(11)**,** 2388-2394. doi: 10.1016/j.chemosphere.2006.04.067.

Seo, J.S., Keum, Y.S., Hu, Y., Lee, S.E., and Li, Q.X. (2007). Degradation of phenanthrene by *Burkholderia* sp. C3: initial 1,2- and 3,4-dioxygenation and meta- and ortho-cleavage of naphthalene-1,2-diol. *Biodegradation* 18(1)**,** 123-131. doi: 10.1007/s10532-006-9048-8.

Sepic, E., and Leskovsek, H. (1999). Isolation and identification of fluoranthene biodegradation products. . *Analyst* 124**,** 1765-1769.

Story, S.P., Parker, S.H., Hayasaka, S.S., Riley, M.B., and Kline, E.L. (2001). Convergent and divergent points in catabolic pathways involved in utilization of fluoranthene, naphthalene, anthracene, and phenanthrene by *Sphingomonas paucimobilis* var. EPA505. *J Ind Microbiol Biotechnol* 26(6)**,** 369-382.

Suzuki, S., and Hiraishi, A. (2007). *Novosphingobium naphthalenivorans* sp. nov., a naphthalene-degrading bacterium isolated from polychlorinated-dioxin-contaminated environments. *J Gen Appl Microbiol* 53(4)**,** 221-228.

Tagger, S., Truffaut, N., and Le Petit, J. (1990). Preliminary study on relationships among strains forming a bacterial community selected on naphthalene from a marine sediment. *Can J Microbiol* 36(10)**,** 676-681.

Takáčová, A., Smolinská, M., Ryba, J., Mackuľak, T., Jokrllová, J., Hronec, P., et al. (2014). Biodegradation of Benzo[a]Pyrene through the use of algae. *CENT EUR J CHEM* 12(11)**,** 1133-1143.

Takizawa, N., Iida, T., Sawada, T., Yamauchi, K., Wang, Y.W., Fukuda, M., et al. (1999). Nucleotide sequences and characterization of genes encoding naphthalene upper pathway of *Pseudomonas aeruginosa* PaK1 and *Pseudomonas putida* OUS82. *J Biosci Bioeng* 87(6)**,** 721-731.

Tao, X.Q., Lu, G.N., Dang, Z., Yi, X.Y., and Yang, C. (2007). Isolation of phenanthrene- degrading bacteria and characterization of phenanthrene metabolites. . *World J Microbiol Biotechnol.* 23**,** 647-654.

Tapilatu, Y.H., Grossi, V., Acquaviva, M., Militon, C., Bertrand, J.C., and Cuny, P. (2010). Isolation of hydrocarbon-degrading extremely halophilic archaea from an uncontaminated hypersaline pond (Camargue, France). . *Extremophiles* 14**,** 225-231.

Tian, L., Ma, P., and Zhong, J. (2003). Impact of the presence of salicylate or glucose on enzyme activity and phenanthrene degradation by *Pseudomonas mendocina*. . *Proc Biochem.* 38**,** 1125-1132.

Toledo, F.L., Calvo, C., Rodelas, B., and Gonzalez-Lopez, J. (2006). Selection and identification of bacteria isolated from waste crude oil with polycyclic aromatic hydrocarbons removal capacities. *Syst Appl Microbiol* 29(3)**,** 244-252. doi: 10.1016/j.syapm.2005.09.003.

Ueno, R., Wada, S., and Urano, N. (2008). Repeated batch cultivation of the hydrocarbon-degrading, micro-algal strain *Prototheca zopfii* RND16 immobilized in polyurethane foam. *Can J Microbiol* 54(1)**,** 66-70. doi: 10.1139/w07-112.

Van Herwijnen, R., Springael, D., Slot, P., Govers, H.A.J., and Parsons, J.R. (2003a). Degradation of anthracene by *Mycobacterium* sp. Strain LB501T proceeds via a novel pathway, through o-phthalic acid. *Appl Environ Microbiol* 69**,** 186-190.

Van Herwijnen, R., Wattiau, P., Bastiaens, L., Daal, L., Jonker, L., Springael, D., et al. (2003b). Elucidation of the metabolic pathway of fluorene and cometabolic pathways of phenanthrene, fluoranthene, anthracene and dibenzothiophene by *Sphingomonas* sp. LB126. *Res Microbiol* 154**,** 199-206.

Vila, J., Lopez, Z., Sabate, J., Minguillon, C., Solanas, A.M., and Grifoll, M. (2001). Identification of a novel metabolite in the degradation of pyrene by *Mycobacterium* sp. strain AP1: actions of the isolate on two- and three-ring polycyclic aromatic hydrocarbons. *Appl Environ Microbiol* 67(12)**,** 5497-5505. doi: 10.1128/AEM.67.12.5497-5505.2001.

Walter, U., Beyer, M., Klein, J., and Rehm, H.J. (1991). Degradation of pyrene by *Rhodococcus* sp. UW1. *Appl. Microbiol. Biotechnol.* 34**,** 671-676.

Wang, B., Lai, Q., Cui, Z., Tan, T., and Shao, Z. (2008). A pyrene-degrading consortium from deep-sea sediment of the West Pacific and its key member *Cycloclasticus* sp. P1. *Environ Microbiol* 10(8)**,** 1948-1963. doi: 10.1111/j.1462-2920.2008.01611.x.

Warshawsky, D., Cody, T., Radike, M., Reilman, R., Schumann, B., LaDow, K., et al. (1995). Biotransformation of benzo[a]pyrene and other polycyclic aromatic hydrocarbons and heterocyclic analogs by several green algae and other algal species under gold and white light. *Chem Biol Interact* 97(2)**,** 131-148.

Warshawsky, D., Radike, M., Jayasimhulu, K., and Cody, T. (1988). Metabolism of benzo(a)pyrene by a dioxygenase enzyme system of the freshwater green alga *Selenastrum capricornutum*. *Biochem Biophys Res Commun* 152(2)**,** 540-544.

Wattiau, P., Bastiaens, L., van Herwijnen, R., Daal, L., Parsons, J.R., Renard, M.E., et al. (2001). Fluorene degradation by *Sphingomonas* sp. LB126 proceeds through protocatechuic acid: a genetic analysis. *Res Microbiol* 152(10)**,** 861-872.

Weissenfels, W.D., Beyer, M., and Klein, J. (1990). Degradation of phenanthrene, fluorene and fluoranthene by pure bacterial cultures. *Appl Microbiol Biotechnol* 32(4)**,** 479-484.

Widada, J., Nojiri, H., Kasuga, K., Yoshida, T., Habe, H., and Omori, T. (2002). Molecular detection and diversity of polycyclic aromatic hydrocarbon-degrading bacteria isolated from geographically diverse sites. *Appl Microbiol Biotechnol* 58(2)**,** 202-209.

Yamazoe, A., Yagi, O., and Oyaizu, H. (2004). Degradation of polycyclic aromatic hydrocarbons by a newly isolated dibenzofuran-utilizing Janibacter sp. strain YY-1. *Appl Microbiol Biotechnol* 65(2)**,** 211-218. doi: 10.1007/s00253-003-1541-y.

Yang, Y., Chen, R.F., and Shiaris, M.P. (1994). Metabolism of naphthalene, fluorene, and phenanthrene: preliminary characterization of a cloned gene cluster from *Pseudomonas putida* NCIB 9816. *J Bacteriol* 176(8)**,** 2158-2164.

Zeinali, M., Vossoughi, M., and Ardestani, S.K. (2008a). Naphthalene metabolism in *Nocardia otitidiscaviarum* strain TSH1, a moderately thermophilic microorganism. *Chemosphere* 72(6)**,** 905-909. doi: 10.1016/j.chemosphere.2008.03.038.

Zeinali, M., Vossoughi, M., and Ardestani, S.K. (2008b). Degradation of phenanthrene and anthracene by *Nocardia otitidiscaviarum* strain TSH1, a moderately thermophilic bacterium. *J Appl Microbiol* 105(2)**,** 398-406. doi: 10.1111/j.1365-2672.2008.03753.x.

Zhang, H., Kallimanis, A., Koukkou, A.I., and Drainas, C. (2004). Isolation and characterization of novel bacteria degrading polycyclic aromatic hydrocarbons from polluted Greek soils. *Appl Microbiol Biotechnol* 65(1)**,** 124-131. doi: 10.1007/s00253-004-1614-6.
